# The minnow *Phoxinus lumaireul* (Leuciscidae) shifts the Adriatic–Black Sea basin divide in the north‐western Dinaric Karst region

**DOI:** 10.1002/eco.2449

**Published:** 2022-07-13

**Authors:** Susanne Reier, Luise Kruckenhauser, Aleš Snoj, Peter Trontelj, Anja Palandačić

**Affiliations:** ^1^ First Zoological Department Natural History Museum Vienna Vienna Austria; ^2^ Department of Evolutionary Biology University of Vienna Vienna Austria; ^3^ Central Research Laboratories Natural History Museum Vienna Vienna Austria; ^4^ Department of Animal Science, Biotechnical Faculty University of Ljubljana Domžale Slovenia; ^5^ Department of Biology, Biotechnical Faculty University of Ljubljana Ljubljana Slovenia

**Keywords:** Dinaric Karst, paleohydrology, *Phoxinus*, underground connections, underground migration

## Abstract

Karst landscapes are characterized by intermittent and sinking streams. The most common method used to study underground hydrological connections in karst is tracing tests. However, a more biologically oriented approach has been suggested: analysis of the genetic structure of aquatic organisms. Biological tracers can be sought among trogloxenes, that is, surface species that occasionally enter caves and groundwater. One such example is the fish genus *Phoxinus*, which exhibits high genetic diversity and complex phylogeography in the Balkan Peninsula. In the north‐western Dinaric Karst, the complex hydrological network was digitalized in 2020. Contemporaneously, *Phoxinus lumaireul* populations in the Slovenian Dinaric Karst were intensively sampled and analysed for fragments of two mitochondrial genes and one nuclear gene. The derived phylogeographic structure and data on hydrological connections were compared to evaluate support for three alternative scenarios: The genetic structure (1) is a consequence of the ongoing geneflow through underground connections, (2) reflects a previous hydrological network or (3) is an outcome of anthropogenic translocations. The results suggest that the first two scenarios seem to have played a major role, while the third has not had profound effects on the genetic composition. Comparison between the genetic structure of Slovenian Dinaric Karst sampling sites and that of hydrologically isolated reference sampling sites indicated a greater genetic connectivity in the former. Moreover, the range of Adriatic (1a) and Black Sea (1c) haplotypes does not correspond to the Adriatic–Black Sea basin divide but is shifted northwards.

## 1 INTRODUCTION

Karst landscapes are formed by soluble carbonate rocks, commonly limestone or dolomite, which, besides possessing specific features (e.g., sinkholes and caves), exhibit a lack of contiguous surface river systems (RS) and are characterized instead by intermittent and sinking streams. However, karst landscape patchy surface waters may be interconnected via complex underground drainage networks, with extreme spatial and temporal oscillations in groundwater levels (Bonacci, 1987; Bonacci & Živaljević, 1993). Several scientific disciplines, for example, hydrology, geology and biology, attempt to understand the intricate structure of karst aquifers in order to support water management, control pollution and foster conservation of endangered species (Ford & Williams, 2007). The most commonly used method for studying underground hydrological connections in karst is the tracing test (Field, 1999) for which tracers are substances (physical, chemical or isotopic) carried (as, e.g., a dye or salt) by water and that provide information on flow direction, rate and velocity (Petrič et al., 2020). Complementary to classical water tracing methods, a more recent, biologically oriented approach has been suggested: analysis of the genetic structure of aquatic organisms inhabiting underlying karstic aquifers (Pipan & Culver, 2007; Verovnik et al., 2003). In addition to revealing links between water bodies, biological connectivity reflects the ecology of the studied organisms and can convey information on pore size, water quality and, by combining active and passive swimmers, alternative flow directions (Humphreys, 2009; Konec et al., 2016; Verovnik et al., 2005).

The Dinaric Karst of the Western Balkan Peninsula is particularly suited for studies comparing genetic and hydrological connectivity, as it comprises among the best‐investigated karst areas in terms of hydrogeology (Bonacci, 1987) and population structure of aquatic fauna associated with subterranean waters (e.g., Bilandžija et al., 2013; Palandačić, Bonacci, & Snoj, 2012; Zakšek et al., 2009). One of the characteristics of Dinaric Karst is the presence of poljes, flat alluvial depressions encircled by higher ground composed of permeable rocks that can be traversed by sinking rivers. Superficial sections of these rivers, which flow partly underground, are the only sizable surface wetlands in karst landscapes in which, as a result, they act as habitat islands.

Thus, in fish, karst has often been considered an isolating factor in organismal dispersal (e.g., Buj et al., 2014; Mustafić et al., 2017; Perea et al., 2010), promoting speciation and consequently increasing the species richness of the area. Similarly, the dissected and patchy karst landscape is considered to have promoted vicariance also in other epigean organisms, for example, various mammals, crayfish and insects (e.g., Klobučar et al., 2013; Krystufek et al., 2007; Previšić et al., 2014) as well as in cave fauna (e.g., Bilandžija et al., 2013; Caccone & Sbordon, 2001).

Nevertheless, it has been suggested that some fish can use underground passages to migrate between isolated, sinking streams (Palandačić, Matschiner, et al., 2012; Snoj et al., 2008; Zupančič, 2008) and some underground migration has also been shown in the epigean isopod *Asellus* (Konec et al., 2016) and amphipods *Echinogammarus* and *Fontogammarus* (Žganec et al., 2016), as well as in subterranean *Proteus* (Zakšek et al., 2018).

Recently, the complex hydrological network of the north‐western Dinaric Karst in Slovenia (hereafter referred to as Slovenian Dinaric Karst) was digitalized by Petrič et al. (2020), who employed more than 200 tracing tests. As a result, the hydrology of the area is fairly well understood: Whereas the western part of the Slovenian Dinaric Karst drains towards the Adriatic Sea, the central and southern parts flow to the Black Sea. Meanwhile, because the water flow fluctuates spatially and temporally (Bonacci, 1987, 2013; Konec et al., 2016), the karstic subterranean watershed separating the basins is difficult to determine (Habič, 1989; Terzić et al., 2014). There are five major RS in Slovenian Dinaric Karst corresponding, respectively, to the main rivers Ljubljanica, Vipava, Reka, Krka and Kolpa (Gams, 1993). The watersheds of all five rivers include both karstic and non‐karstic areas, as well as several underground connections among them.

Biological tracers revealing subterranean flows can be sought among obligate cave species or among trogloxenes, that is, surface species that occasionally enter caves and groundwater, where they are able to survive but not reproduce (Howarth & Moldovan, 2018). Trogloxenes seem to be especially suitable as indicator species, because they connect different isolated surface wetlands such as karst poljes, as well as karstic and non‐karstic areas. One such example is the group of ubiquitous small minnows of the genus *Phoxinus* Rafinesque, 1820 (formerly in the family Cyprinidae, now in Leuciscidae; Schönhuth et al., 2018) that inhabit diverse habitats, from mountain streams to lowland rivers and lakes (Frost, 1943; Tack, 1940). Recent genetic studies of *Phoxinus* spp. in Europe have revealed a high level of genetic diversity and complex phylogeography in the Balkan Peninsula (Palandačić et al., 2015; Palandačić et al., 2017; Vučić et al., 2018) that do not follow zoogeographic patterns defined by drainage boundaries (e.g., Konec et al., 2016; Palandačić et al., 2017; Palandačić et al., 2020; Zogaris et al., 2009). This distribution of *Phoxinus* genetic lineages and the admixture detected among them was ascribed to human‐mediated actions (see, e.g., Museth et al., 2007; Miró & Ventura, 2015; Knebelsberger et al., 2015). However, it has also been suggested that, besides human introductions (Vučić et al., 2018), the admixture of lineages in the Western Balkans can also be attributed to natural causes, occurring as a consequence of historical and recent migration patterns of minnows (Palandačić et al., 2015, 2020). These effects also apply to the species *Phoxinus lumaireul* (Schinz, 1840) (clade 1 sensu Palandačić et al., 2017), which inhabits the Adriatic and the Black Sea basins from Italy via Slovenia, Croatia, Austria and Bosnia‐Herzegovina to Serbia and which harbours notable genetic diversity with six distinct genetic lineages (subclades 1a–f sensu Palandačić et al., 2017).

The combination of an organism, such as *P. lumaireul*, with high levels of genetic diversity inhabiting a hydrological system and a complex but well studied system of subterranean water connections (Slovenian Dinaric Karst) provides an excellent research opportunity for deciphering the factors that might shape phylogeographic patterns and biological diversity of surface‐dwelling species in karstic areas. Thus, *P. lumaireul* populations in Slovenian Dinaric Karst were intensively sampled and analysed for partial fragments of two mitochondrial genes (*cytochrome b* and *cytochrome c oxidase subunit 1*) and one nuclear gene (*ribosomal protein S7*). The derived phylogeographic structure, along with data on past and present hydrological connections, was compared in order to assess support for three alternative scenarios: that the genetic structure (1) is a consequence of the ongoing geneflow through underground connections, (2) reflects the past hydrological network or (3) is an outcome of anthropogenic translocations.

## 2 MATERIAL AND METHODS

### 2.1 Study area and grouping of samples

**FIGURE 1:**
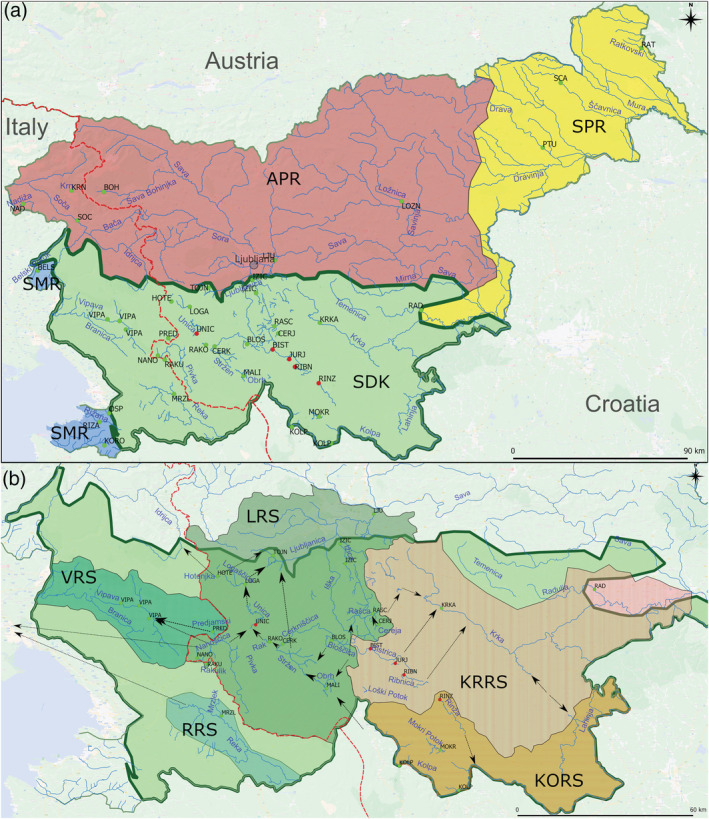
North‐western Dinaric Karst and adjacent regions. (a) Zoogeographic regions of Slovenia including sampling sites. Names of main rivers of Slovenia are given. Abbreviations: APR, Alpine and Prealpine Regions; SDK, Slovenian Dinaric karst; SMR, Submediterranean region; SPR, Subpannonian region. (b) Major River systems in SDK. Arrows represent underground connections between rivers. Red dashed line represents divide between Adriatic and Black Sea basins. Abbreviations: VRS, Vipava river system; RRS, Reka river system; LRS, Ljubljanica river system; KRRS, Krka river system; KORS, Kolpa river system. Green dots represent sites were *Phoxinus* was present; red dots, sampling sites that were not inhabited by *Phoxinus*.

In concordance with several abiotic properties (e.g., tectonic, lithological, relief, climatic and edaphic), Slovenia can be divided into five zoogeographic regions (Figure 1a): Slovenian Dinaric Karst (SDK), Submediterranean Region (SMR), Subpannonian Region (SPR) and Alpine and Prealpine Region (herein merged into one group, APR) (Mršić et al., 1997). The main part of the karst area of Slovenia comprises the Dinaric and Alpine regions but also partly the Prealpine and SMRs (Mihevc et al., 2016).

The main study area was SDK, which included 20 sampling sites. Additionally, there were five sampling sites in APR, four in SMR and three in SPR, adopted from previous studies and included as reference sampling sites.

The sampling sites in SDK were sub‐divided into five groups corresponding to five RS: (1) Ljubljanica RS (LRS), (2) Vipava RS (VRS), (3) Reka RS (RRS), (4) Krka RS (KRRS) and (5) Kolpa RS (KORS) (Figure 1b). LRS includes 13 sampling sites (MALI, CERK, RAKO, NANO, RAKU, HOTE, LOGA, TOJN, IZIC, BLOS, RASK, CERJ, LJU), VRS two sites (PRED, VIPA), RRS one site (MRZL), KRRS two sites (KRKA, RAD) and KORS two sites (KOLP, MOKR); see Table 1 and Figure 1b.

**TABLE 1:** *Phoxinus lumaireul* sampling sites from Slovenia

ID	Sampling site	Drainage	Clade–group	Number		
				Present study	Palandačić et al. (2015, 2017)	Total
Slovenian Dinaric Karst						
*Ljubljanica river system (LRS)*						
BLOS	Bloščica	B	1c	29	4	33
CERJ	Cereja	B	1c	36	0	36
CERK	Cerkniščica	A, B	1a/1c	28	8	36
HOTE	Hotenjka	B	1a	19	0	19
IZIC	Izica	B	1c (1a)	25	0	25
LJU	Ljubljanica River	B	1c	0	2	2
LOGA	Logaščica	B	1a	20	0	20
MALI	Mali Obrh	A, Danubian	1a (1c)	19	6	25
NANO	Nanoščica	A, B	1a	42	6	48
RAKO	Rak	B	1a (1c)	19	0	19
RAKU	Rakulik	B	1a	26	0	26
RASC	Raska	B	1c (1a)	17	0	17
TOJN	Tojnica	B	1c (1a)	18	0	18
*Reka river system (RRS)*						
MRZL	Stream Mrzlek	A	1a	25	4	29
*Vipava river system (VRS)*						
PRED	Lovka	A	1a	33	6	39
VIPA	Vipava and tributaries	A	1a	10	2	12
*Kolpa river system (KORS)*						0
KOLP	Kolpa	B	1b	26	6	32
MOKR	Mokri Potok	B	1b	34	0	34
*Krka river system (KRRS)*						
KRKA	Krka	B	1c	33	0	33
RAD	Radulja	B	1c	0	10	10
						513
Alpine & Prealpine Regions						
BOH	Bohinj Lake	B	1c	0	5	5
KRN	Lake Krn	A	1a	0	4	4
LOZN	Ložnica	B	1c	3	3	6
NAD	Nadiža	A	1a	0	2	2
SOC	Soča	A	1a	0	1	1
						18
Submediterranean Region						
BELS	Belski Potok	A	1a	6	5	11
KORO	Malinska	A	1a	3	4	7
OSP	Osapska	A	1a	0	2	2
RIZA	Rižana	A	1a	6	5	11
						31
Subpannonian Region						
PTU	Drava	B	1d	0	5	5
RAT	Ratkovski	B	1d	0	4	4
SCA	Ščavnica	B	1d	0	7	7
						16
Summary				477	101	578

*Note*: Sampling sites, belonging to the Adriatic (A) or Black Sea (B) basins (drainages), clade–group affiliation, number of examined specimens per location (and subdivision of number of specimens from this study and from previous studies (Palandačić et al., 2015, 2017) per location) and total number are reported. Clade–group is reported in parentheses when only 1 or 2 individuals from the sampling site belong to this clade–group

In the APR, there were five sampling sites (BOH, KRN, LOZN, NAD, SOC), in the SMR four sites (BELS, KORO, OSP, RIZA) and in the SPR, three sampling sites (PTU, RAT, SCA).

In comparison to the reference sampling sites, those of SDK exhibit high hydrological connectivity (Petrič et al., 2020). As the hydrological system is very complex and the local names of the rivers might be challenging for nonnative speakers, a detailed description of the sampled streams and their mutual underground connections is presented in the Supporting Information, while a simplified presentation of the main interconnections is depicted in Figure 1b. None of the reference sampling sites is connected underground to the SDK sampling sites (Figures 1b and S1).

### 2.2 Sampling design

The data from Petrič et al. (2020) are deposited at the website of the Slovenian Environment Agency (http://gis.arso.gov.si/) and were downloaded and plotted in QGIS 3.12.2 (QGIS Development Team, 2009). The division of the sampling sites as described above was drawn manually in QGIS (depicted in Figure 1) and plotted together with the underground connections in Figure S1. A simplified depiction of the underground connections is shown in Figure 1b.

In total, 477 specimens of *P. lumaireul* were collected between the years 2016 and 2020 from 22 sampling sites (Figure 1, Table 1) using electrofishing, fishing nets and traps. Besides the freshly collected samples, the dataset also includes 101 previously published sequences (Palandačić et al., 2015, 2017) from 10 additional sampling sites. The combined dataset of new and GenBank sequences comprises 578 sequences (for respective GenBank accession numbers see Table S1).

### 2.3 Mitochondrial DNA

#### 2.3.1 DNA extraction, amplification, sequencing and alignment

Total DNA was extracted from 477 fin clips in a clean room using QIAmp DNeasy Blood and Tissue Kit (QIAGEN, Hilden, Germany) and following the manufacturer's protocol. Partial fragments of the mitochondrial (mt) *cytochrome c oxidase subunit 1* (*COI*) gene (the so‐called barcoding region) and the mt *cytochrome b* (*cytb*) gene were amplified using the primer pairs FishF1/FishR1 (*COI*; Behrens‐Chapuis et al., 2015) and GluF/ThrR (*cytb*; Bergsten et al., 2014) with the polymerase chain reaction (PCR) protocol described in (Palandačić et al., 2017). Sequencing was performed at Microsynth (Balgach, Switzerland) in both directions using the PCR primers.

Sequences were manually checked, trimmed and unambiguously aligned (no missing data) in Geneious v2.10.3 (https://www.geneious.com). *COI* and *cytb* sequences were subsequently concatenated using Geneious, and the concatenated alignment, including available sequences from GenBank, was used for all further genetic analysis (hereinafter referred to as combined *COI*–*cytb*).

#### 2.3.2 Phylogenetic tree reconstruction

Sequences of *COI*–*cytb* were collapsed to unique haplotypes using FaBox v1.5 (Villesen, 2007). The best‐fit models (K2P+G4 for *COI*; GTR+F+G4 for *cytb*) were selected using ModelFinder (Kalyaanamoorthy et al., 2017) applying the BIC criterion. Phylogenetic relationships were inferred using maximum likelihood (ML) and Bayesian inference (BI) performed in RAxML‐HPC v8 (Stamatakis, 2014) and Mr. Bayes v3.2 (Ronquist et al., 2012), respectively. We applied the GTR+G model for ML tree search with 10,000 bootstrap replicates to assess nodal support. BI was performed with three runs, each with four chains, and run for 50,000,000 generations. Trees and parameters were sampled every 1000 generations. After inspecting effective sample size (ESS) parameters that exceeded 300 in Tracer v1.7.1 (available at http://tree.bio.ed.ac.uk/software/tracer/), the first 25% of trees were discarded as burn‐in, and a 50% majority rule consensus tree was built from the remaining trees.

#### 2.3.3 Median‐joining network, population diversity indices

The median‐joining haplotype network (Bandelt et al., 1999) was calculated for *COI*–*cytb* using the software PopART v1.7 (Leigh & Bryant, 2015). Haplotype diversity (*Hd*), nucleotide diversity (*π*), number of polymorphic sites (*S*) and mean number of pairwise differences (*k*) were calculated using DnaSP v6 (Rozas et al., 2017).

#### 2.3.4 Pairwise distances

Uncorrected pairwise (p‐)distances were calculated using MEGA 10.0.5 (Kumar et al., 2018) and plotted using ggplot2 v3.3.5 (Wickham, 2016) in R v3.6.3 (R Core Team, 2020).

#### 2.3.5 Spatial population clustering and analysis of molecular variance (AMOVA)

In order to gather information on the spatial distribution of populations, cluster analysis was performed with GENELAND v4.9.2 (Guillot et al., 2005; Guillot et al., 2008) in R v3.6.3 (R Core Team, 2020) using the *COI–cytb* dataset. GENELAND enables the detection and location of genetic discontinuities between populations and the correlation of these discontinuities with landscape and environmental features (Guillot et al., 2005). The number of populations (K) was allowed to vary between 1 and 20. Twenty independent runs were conducted, each with 1,000,000 Markov Chain Monte Carlo (MCMC) iterations and with sampling at every 100 steps. The maximum number of nuclei in the Poisson–Voronoi tessellation was fixed to 1665 (full GENELAND, see below) and 1500 (sub‐set GENELAND), and 1000 iterations were discarded as burn‐in.

The GENELAND analysis was run on two levels: 1, full GENELAND, including all sampling sites from SDK, APR, SMR and SPR; and 2, sub‐set GENELAND, including SDK sampling sites only in order to gain a better insight into the fine‐scale population structure within this area.

To test whether the distribution of genetic variance corresponds with the clustering of populations suggested by GENELAND, AMOVA (Excoffier et al., 1992) was conducted using Arlequin v3.5.2.2 (Excoffier & Lischer, 2010) for both full and sub‐set GENELAND analysis. The proportion of genetic variation found among sampling sites (F_ST_), among sampling sites within groups (F_SC_) and among groups (F_CT_), was estimated. Significance associated with the fixation indices was evaluated through random permutation procedures (10,000 permutations).

#### 2.3.6 Divergence time dating

In order to estimate the timing of the splits between and within the mt‐clades of the genus *Phoxinus*, divergence time dating was performed based upon the mt‐*cytb* phylogenetic tree. While divergence time estimation inferred from phylogenetic trees is frequently applied (Hedges et al., 2015), the results are often questioned (Bromham & Woolfit, 2004) and exhibit large confidence intervals (Warnock et al., 2017). To ensure reliable divergence time estimates, *cytb* was chosen because the evolutionary rate has already been estimated for Plagopterinae (a subfamily of Leuciscidae), which contains the true minnows including *Phoxinus* (Dowling et al., 2002). Second, outgroups were selected on the basis of the phylogenies of the complete mt genome published in Imoto et al. (2013) and a recently published study of Schönhuth et al. (2018). All sequences used are reported in Table S2 along with the corresponding Genbank numbers. Finally, fossil data were used as additional calibrating points to increase the accuracy of the evolutionary rate estimation, and the chosen model was tested by nested sampling (Russel et al., 2019) to check the reliability of the results (Ritchie et al., 2016).

The trees were constructed using BEAST2 v2.5 (Bouckaert et al., 2019). The chosen site model was GTR+F+G4, selected using ModelFinder (Kalyaanamoorthy et al., 2017) under the Bayesian information criterion (BIC). The tree was time‐calibrated based on the estimated divergence times of Cyprininae (68–97.1 Mya, Saitoh et al., 2011), Leucisinae (23.5–24.5 Mya, Böhme & Ilg, 2003), *Chondrostoma* (5.21 Mya, Böhme & Ilg, 2003), *Squalius* (6.56 Mya, Böhme & Ilg, 2003) and *Phoxinus* (29.2–30.2 Mya, Mödden et al., 2000). A lognormal distribution with an offset of the minimum age was used, and the standard deviation adjusted so that the above‐stated divergence time span was covered by 95% of the sampled divergence date priors (Drummond et al., 2006; Ho, 2007). As an additional calibration point, an uncorrelated, relaxed log‐normal clock, corresponding to an evolutionary rate of 0.0053 mutations per site per million years, was applied, a rate generally used for the Leuciscidae family (Dowling et al., 2002). Node times were estimated under a birth‐death tree prior, which is often used when extinction rates are considered negligible and which has been shown to produce stable results (Ritchie et al., 2016). The analysis was run for 50,000,000 generations and sampled every 1000 generations. For further support of the model, nested sampling analyses (Russel et al., 2019) were applied in BEAST2 to compare the marginal likelihood of the above‐described model and a strict clock model and different tree priors (coalescent Bayesian skyline and Yule model, detailed information can be found in Table S3). Forty‐five particles for all models were used to compute the marginal likelihood and its standard deviation. Then, the marginal likelihoods of the models were compared and the log Bayes factor calculated.

The log‐file of the final model was inspected in Tracer v1.7.1 and showed large values (>300) for the ESS. A maximum clade credibility (MCC) tree was estimated using Tree Annotator (Drummond & Rambaut, 2007). The final tree was plotted against the geological timescale using the R package strap v1.4 (Bell & Lloyd, 2014) in R v3.6.3 (R Core Team, 2020).

### 2.4 Nuclear DNA

#### 2.4.1 DNA amplification, cloning, sequencing and alignment

The usefulness of the nuclear (nc) markers *recombination activating gene* 1 (*RAG1*) and *internal transcribed spacer* 1 (*ITS1*) for the detection of hybrids within the genus *Phoxinus* has been questioned previously (Palandačić et al., 2020). Thus, another nuclear partial fragment, the first intron of the single‐copy *nuclear ribosomal protein S7* (*RPS7*) gene (used in other phylogenetic studies, e.g., Perea et al., 2010) was chosen for analysis to test whether admixture within *P. lumaireul* can be recognized. For amplification, primers S7RPEX1F/S7RPEX2R (*RPS7*; Chow & Hazama, 1998) were used. The PCR mixture had a final volume of 50 μl containing 27.75 μl nuclease‐free water, 1× PCR buffer, 1.5 mM MgCl2, 0.2 mM of each dNTP, 0.2 μM of each primer and 1.25 U/μl AmpliTaq Gold® DNA Polymerase. PCR conditions were as follows: 95°C for 4 min, 35 cycles (94°C for 30 s, 55°C for 30 s, 72°C for 50 s) and final elongation at 72°C for 7 min. Sequencing was performed at Microsynth (Balgach, Switzerland) in both directions using the PCR primers.

After sequencing, heterozygous individuals were detected. However, due to length polymorphism, their sequences could not be read. Therefore, PCRs of 10 random individuals per sampling site (where available) were repeated using the proofreading Phusion® High‐Fidelity Polymerase (New England Biolabs, Ipswich, UK) chosen due to its low error rate (Hestand et al., 2016). The PCR conditions were as follows: 98°C for 30 s, 35 cycles (98°C for 10 s, 64°C for 30 s, 72°C for 30 s) and final elongation for 10 min at 72°C. PCR fragments were purified by adding 2 μl ExoSAP (ExoSAP‐IT; Amersham Biosciences, Arlington Heights, IL) into 5 μl of the PCR product to remove excess primers and dNTPs and cloned with the TOPO‐TA© cloning kit (Invitrogen, Carlsbad, CA, USA). Six clones per individual were sequenced in both directions using M13 universal primers.

Subsequent to cloning, multiple, repeated haplotypes of RPS7 within an individual were excluded from further analysis. The sequences of RPS7 exhibited different lengths, and therefore, an alignment including gaps was produced using the MAFFT algorithm (Katoh & Standley, 2013; fasta file available in the Supporting Information). Simple indel coding was applied in FastGap (Borchsenius, 2009) following the approach of Palandačić et al. (2020).

#### 2.4.2 Phylogenetic tree reconstruction

A phylogenetic tree for *RPS7 was constructed using both BI and ML as described for the mtDNA analysis*. *ModelFinder* (Kalyaanamoorthy et al., 2017) *was used to select the best‐fit model (F81+F+I+G4) using the BIC criterion*.

#### 2.4.3 Median‐joining network, population diversity indices

The median‐joining allele network for *RPS7* and population diversity indices (Hd, π, S, k) were calculated as described for mtDNA analysis.

#### 2.4.4 Pairwise distances

Uncorrected p‐distances were calculated as described for mtDNA analysis.

## 3 RESULTS

### 3.1 Mitochondrial DNA

#### 3.1.1 DNA extraction, amplification, sequencing and alignment

A total of 651 base pairs (bp) of the *COI* and 1091 bp of the *cytb* gene were resolved with sequence analysis of 458 individuals. Together with the sequences from previous studies, the combined *COI–cytb* dataset final alignment included 555 sequences that were 1742 bp in length.

#### 3.1.2 Phylogenetic tree reconstruction

The complete *COI–cytb* dataset was collapsed to 133 unique haplotypes. Both the ML tree and BI tree resulted in a similar topology, though the BI tree had higher statistical support (Figure S2A,B).

In previous research, four genetic lineages were recognized for *P. lumaireul* in Slovenia: 1a, 1b, 1c and 1d (see Introduction); this numbering was also adopted in the present study. However, while the calculated tree supported the clustering of the newly sampled haplotypes to one of the three clades 1a, 1b and 1d, the rest of the haplotypes were not monophyletic but rather formed several sub‐clades. The mt‐clades are named mt‐1a, mt‐1b and mt‐1d, while mt‐1c is referred to as a group, to emphasize its possibly polytomous origin.

**Clade mt‐1a** included samples from the APR (KRN, NAD, SOC) and SMR (OSP, RIZA, KORO, BELS).

Of the SDK samples, RRS (MRZL) VRS (VIPA and PRED) and south‐western LRS (NANO, RAKU, HOTE, LOGA, MALI, RAKO and CERK, which are hydrologically allocated to the Black Sea basin) clustered to this clade. Of 13 LRS sampling sites, seven clustered to clade mt‐1a.

**Clade mt‐1b** included only two sampling sites belonging to the KORS (KOLP and MOKR) in the SDK.

**Group mt‐1c** consisted of sampling sites from the APR (BOH, LOZN) and the SDK samples from KRRS (KRKA, RAD), as well as north‐eastern LRS (BLOS, CERK, CERJ, IZIC, LJU, RASC and TOJN).

Sampling site CERK (LRS) exhibited haplotypes from clade mt‐1a and group mt‐1c; 30 samples bore mt‐1a and six, mt‐1c haplotypes. In IZIC, TOJN and RASC, only one specimen at each sampling site exhibited the mt‐1a haplotype, while the others bore mt‐1c haplotypes. Thus, for the discussion, CERK was grouped with the south‐western LRS sampling sites and the other three to the north‐eastern LRS sampling sites (for details see Median‐joining network).

**mt‐1d** was the most distinctive clade and included samples from the SPR (PTU, RAT, SCA).

#### 3.1.3 Median‐joining network, population diversity indices

**FIGURE 2:**
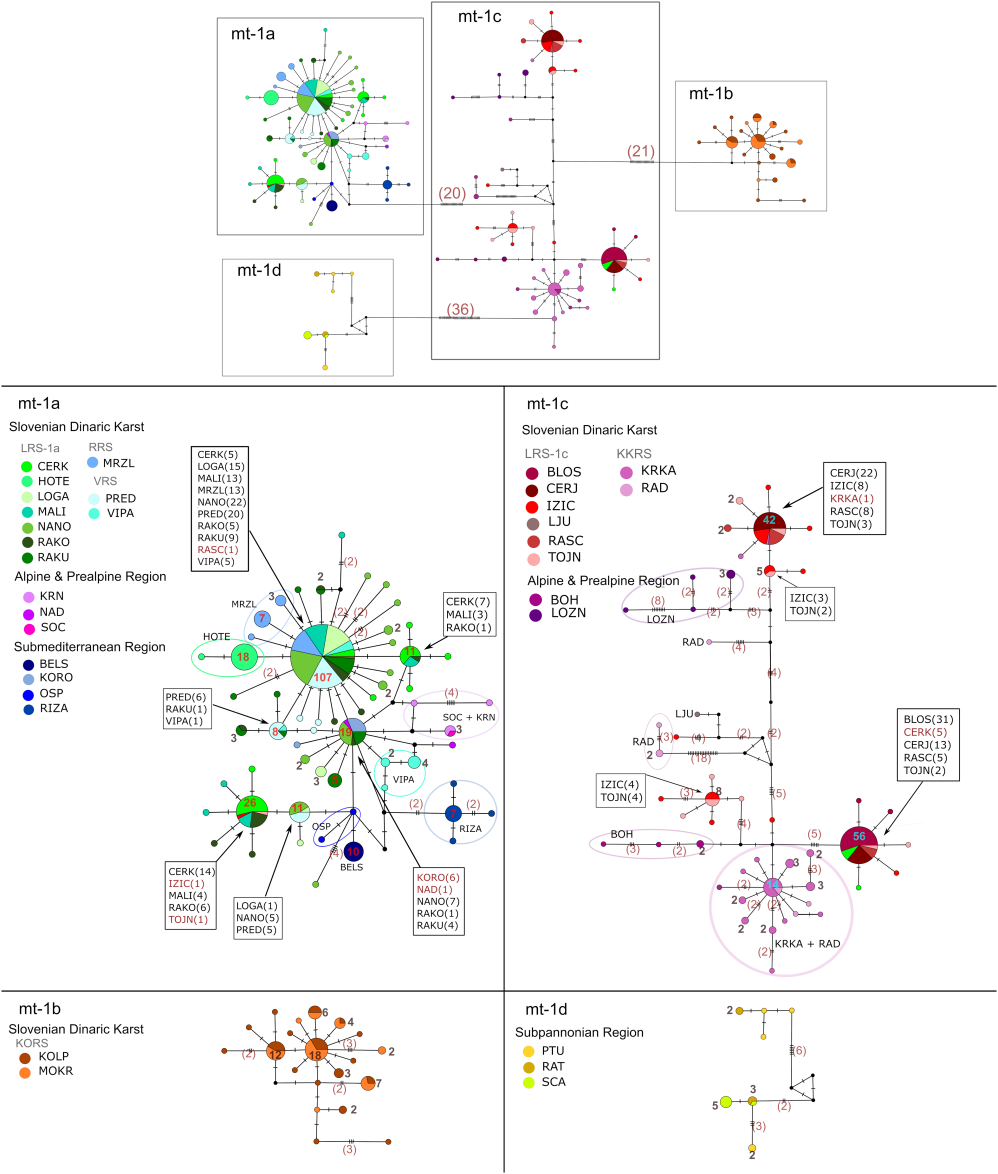
Overview of mitochondrial (*COI‐cytb*) median‐joining haplotype network of *Phoxinus lumaireul* in Slovenia. The dataset includes sequences generated in this study and sequences obtained from previous studies (Palandačić et al., 2015, 2017). A total of 134 unique haplotypes are present. Single clades are presented with a high magnification. Mutation steps are indicated with vertical lines. Number of individuals (when N ≥ 1) contributing to each haplotype are given next to the haplotype. Black dots represent haplotypes missing in the sampling study. Abbreviations of localities are explained in Table 1.

The haplotype network reflected the structure of the phylogenetic tree with clearly separated clades mt‐1a, mt‐1b and mt‐1d. The haplotypes denoted as the group mt‐1c were located in the centre of the network, forming several haplogroups and including single haplotypes (Figure 2). Numbers for haplotype diversity (*Hd*), nucleotide diversity (*π*), number of polymorphic sites (*S*) and mean number of pairwise differences (*k*) are reported in Table 2.

**TABLE 2:** Genetic diversity parameters of the concatenated mitochondrial dataset

ID	N	*Hd*	*π*	*S*	*k*	No. of sequences
Alpine & Prealpine Regions						
BOH	3	0.833	0.00163	5	2.83333	4
KRN	3	0.833	0.00191	6	3.33333	4
LOZN	4	0.8	0.00329	14	5.73333	6
Slovenian Dinaric Karst						
*Ljubljanica river system (LRS)*						
BLOS	3	0.154	0.00007	2	0.12121	33
CERJ	2	0.481	0.00414	15	7.21008	36
CERK	10	0.797	0.00613	38	10.67937	27
HOTE	2	0.00846	0.00006	1	0.10526	19
IZIC	12	0.866	0.00725	59	12.63043	24
LOGA	4	0.432	0.00066	5	1.14211	20
MALI	6	0.656	0.00131	10	2.28458	23
NANO	12	0.744	0.00095	17	1.65217	47
RAKO	10	0.854	0.00167	12	2.91228	19
RAKU	9	0.823	0.00095	9	1.64667	26
RASC	4	0.675	0.00602	40	10.48333	17
TOJN	10	0.922	0.00804	50	14.01307	18
*Reka river system (RRS)*						
MRZL	4	0.668	0.00047	3	0.81053	29
*Vipava river system (VRS)*						
PRED	6	0.617	0.00073	6	1.27094	39
VIPA	5	0.782	0.00106	5	1.84615	12
*Kolpa river system (KORS)*						
KOLP	17	0.933	0.00146	20	2.54637	32
MOKR	8	0.815	0.00112	11	1.94474	34
*Krka river system (KRRS)*						
KRKA	14	0.856	0.00214	33	3.72727	33
RAD	7	0.944	0.00907	39	15.80556	10
Submediterranean Region						
BELS	3	0.4725	0.00084	4	1.46154	10
KORO	2	0.286	0.00016	1	0.28571	7
RIZA	4	0.533	0.00046	4	0.8	6
Subpannonian Region						
PTU	4	0.9	0.00471	14	8.2	5
RAT	2	0.667	0.00421	11	7.33333	4
SCA	2	0.333	0.00019	1	0.33333	8
mt‐1a	59	0.846	0.00152	89	2.64734	295
mt‐1b	20	0.876	0.00128	24	2.23776	66
mt‐1c	48	0.84	0.00606	99	10.55646	179
mt‐1d	7	0.857	0.00362	16	6.30476	15
Summary	133	0.939	0.01253	215	21.8324	555

Abbreviations: *π*, nucleotide diversity; *k*, mean number of pairwise differences; *Hd*, haplotype diversity; N, number of haplotypes; *S*, number of polymorphic sites.

**Clade mt‐1a** consisted of 59 haplotypes and showed a radial structure. It exhibited a relatively high level of *Hd* (*Hd* = 0.85), though the nucleotide diversity (*π* = 0.00152) was low (Table 2).

Clade mt‐1a included the SDK sampling sites, RRS (MRZL), VRS (PRED, VIPA) and the south‐western LRS (NANO, RAKU, HOTE, LOGA, MALI, RAKO). Besides, 30 specimens from CERK and one single specimen from IZIC, RASC and TOJN (north‐western LRS) grouped in mt‐1a. Sampling sites from APR (KRN, NAD, SOC), as well as from SMR (BELS, OSP, RIZA, KORO), clustered in this clade.

The dominant haplotype, representing the centre of the major haplogroup, was shared by 107 individuals (Figure 2) and included specimens from only certain parts of the SDK: the south‐western LRS (CERK, HOTE, MALI, NANO, RAKO, LOGA, RAKU, RASC), RRS (MRZL) and VRS (VIPA, PRED). Five additional haplogroups with more than 10 individuals from different SDK sampling sites were detected: (i) LOGA + NANO + PRED (LRS and VRS), (ii) CERK + MALI + RAKO (only LRS), (iii) a haplogroup formed by CERK + RAKO + MALI, where also the single specimens from TOJN and IZIC cluster (LRS) and (iv) PRED + RAKU + VIPA (LRS and VRS). There was one mixed haplogroup, which, besides the LRS sampling sites NANO + RAKO + RAKU, included also individuals from the SMR (KORO) and APR (NAD).

Besides KORO and NAD, which clustered with some of the SDK sampling sites, other sampling sites from the APR (SOC + KRN) and SMR (BELS, OSP, RIZA) formed their own, exclusive haplogroups. Of the LRS, sampling site HOTE formed its own haplogroup.

**Clade mt‐1b** consisted of 21 haplotypes and exhibited a high level of *Hd* (*Hd *= 0.876), whereas the nucleotide diversity was low (*π* = 0.00128). This clade included sampling sites KOLP and MOKR of the SDK KORS. They shared five most abundant haplotypes, while they also exhibited unique haplotypes (MOKR, 3; KOLP, 13; Figure 2).

**Group mt‐1c** consisted of 48 haplotypes with separated haplogroups and included several very distant haplotypes represented by only one individual. The group showed high levels of haplotype and nucleotide diversity but also a larger number of polymorphic sites compared to the clades (*Hd* = 0.84; *π* = 0.0061; *S* = 99; but see Table 2).

The two biggest haplogroups were formed by individuals from SDK: The first haplogroup consisted of north‐eastern LRS sampling sites BLOS, CERK, CERJ, RASC and TOJN, and the second, north‐eastern LRS sampling sites CERJ, IZIC, RASC and TOJN but including also KRRS sampling site KRKA. These two groups possessed 21 mutational steps between them. Some LRS individuals from IZIC and TOJN also formed two distinct haplogroups, in different parts of the mt‐1c network: one including eight and the other five individuals. Another big haplogroup (with surrounding haplotypes) was formed by samples from the KRRS KRKA + RAD. Samples BOH and LOZN from the APR formed their own haplogroups (with a few haplotypes each).

**Clade mt‐1d** consisted of seven haplotypes, had high levels of haplotype and nucleotide diversity (*Hd* = 0.86; *π* = 0.00362) and was formed exclusively of samples from the SPR.

#### 3.1.4 Pairwise distance matrix (*COI–cytb*)

Among the three clades and the mt‐1c group, clade mt‐1d of the SPR was most distant (2–3%).

**FIGURE 3:**
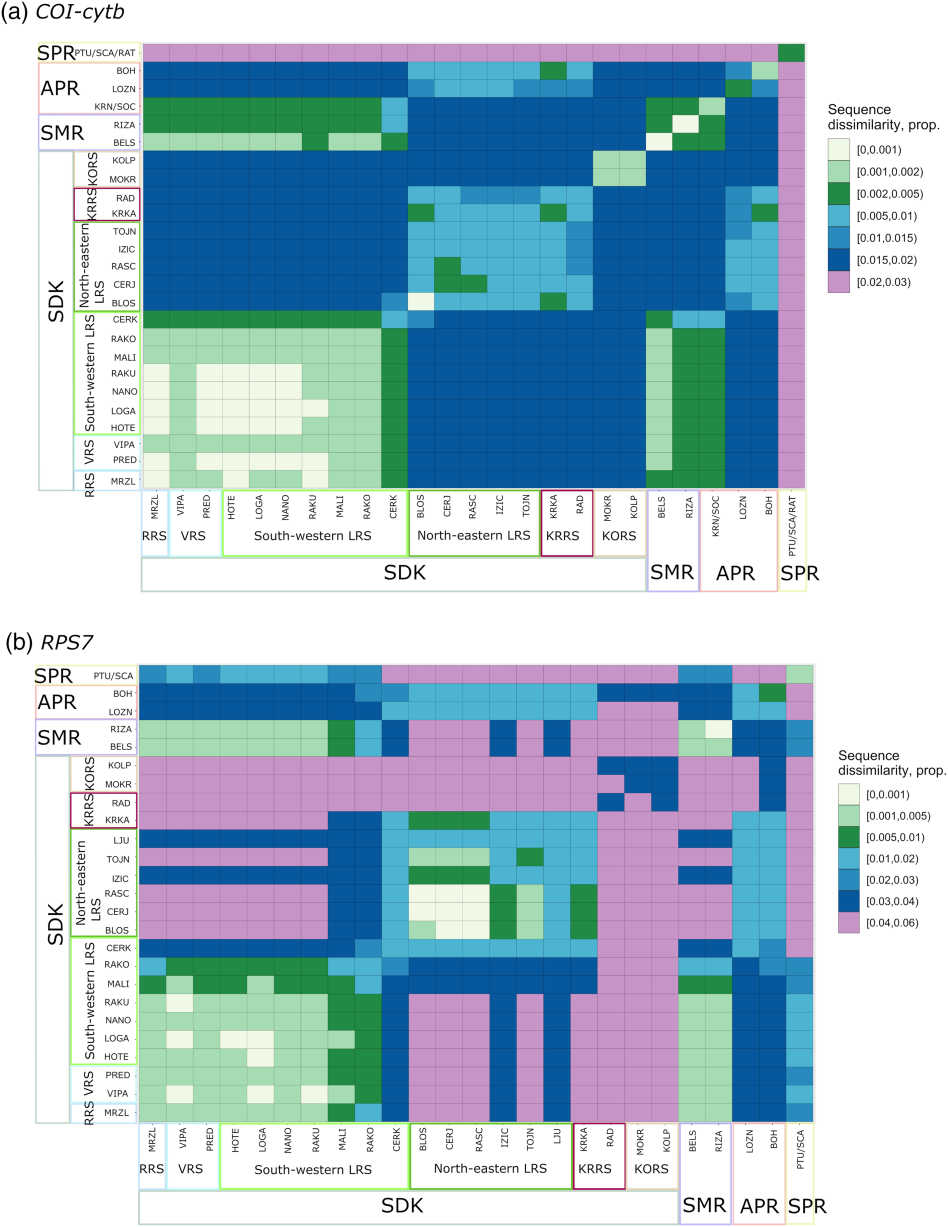
Matrix of uncorrected pairwise p‐distances between sampling sites of *Phoxinus lumaireul* in Slovenia. Distances are pooled to intervals. (a) Mt DNA (*COI‐cytb*). (b) Nc DNA (*RPS7*)

Within the clade mt‐1a, the genetic distances between the SDK and other sampling sites were up to 2% percent (Figure 3a), while the SDK sampling sites (within their respective clade) exhibited genetic distances of up to 0.2%. The only exception was CERK (0.2–0.5%), which bore haplotypes from mt‐1a and mt‐1c.

Within the group mt‐1c, the genetic distances between the SDK sampling sites were up to 1.5%.

The two sampling sites of the KORS exhibited the largest divergence compared to all other SDK sampling sites (1.5–2%).

#### 3.1.5 Spatial population clustering and AMOVA

The genetic groups detected in GENELAND analysis were denoted as ‘clusters’, abbreviated to C for the full GENELAND and SDK‐C for sub‐set GENELAND.

##### Full GENELAND

**FIGURE 4:**
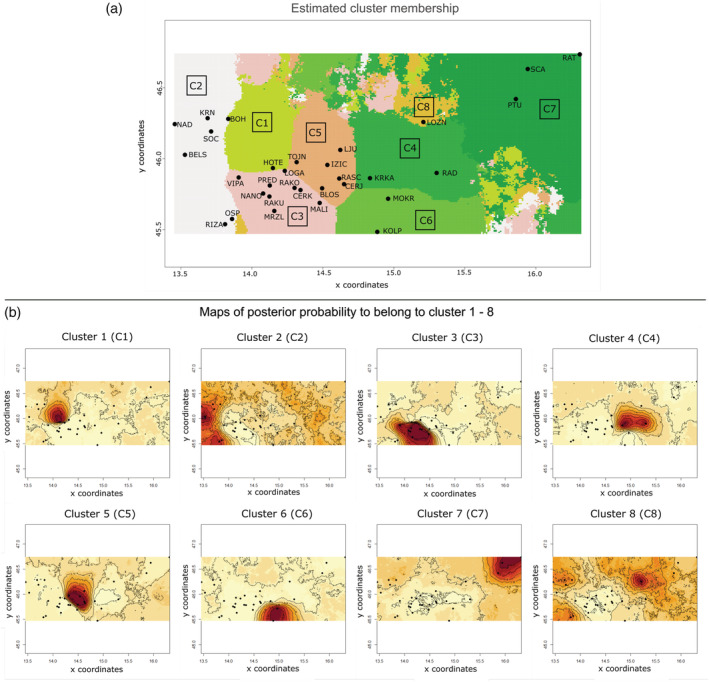
Population Bayesian cluster analysis (GENELAND). Maps showing geographic distribution of sampling sites (black points). (a) Map of cluster membership for each sampling site (K = 8). (b) Relative posterior probability of belonging to each of the eight inferred groups. The darker colour reflects a higher posterior probability.

All 20 runs conducted revealed the same eight clusters. However, in four of 20 runs, single sampling sites from the APR either formed its own cluster (BOH) or clustered to C7 (LOZN) or both. Figure 4 shows the output of the model with the highest log posterior probability. The eight clusters (C1–C8) are presented in Figure 4a, while posterior probabilities for each sampling site belonging to a given cluster are depicted in Figure 4b.

The sampling sites from the APR and SMR clustered together (C2), as did those from the SPR (C7), while LOZN, located in the APR, formed a distinct cluster (C8). The clustering of the SDK sampling sites was stable in all runs and revealed the following clusters: C1 comprised the sampling site HOTE (south‐western LRS), C3 included the sampling sites from VRS, RRS and south‐western LRS, C4 the KRRS, C5 the north‐eastern LRS and C6, the KORS.

Overall, genetic differentiation among the clusters revealed by GENELAND was significant (AMOVA F_ST_ = 0.853; P ≤ 0.0001; Table 4). The results indicated that 78.17% of the variation was among the clusters and 14.74% within sampling sites (P ≤ 0.0001, Table 3).

**TABLE 3:** Analysis of molecular variance (AMOVA) and degrees of freedom (d.f.) for COI‐cytb sequences in all sampling sites of the present study (full GENELAND) and in the samples located in Slovenian Dinaric karst (sub‐set GENELAND)

Source of variation	d.f.	Sum of squares	Variance components	Percentage variation	Fixation indices	P values
Full GENLAND						
Among clusters	7	4531.898	10.80656	78.17	F_CT_ = 0.78171	<0.0001
Among sampling sites within clusters	22	434.713	0.97991	7.09	F_SC_ = 0.32471	<0.0001
Within sampling sites	524	1067.837	2.03786	14.74	F_ST_ = 0.85259	<0.0001
Total	553	6034.448	13.82432			
Sub‐set GENELAND						
Among clusters	4	3804.980	11.461	79.94	F_CT_ = 0.79937	<0.0001
Among sampling sites within clusters	15	294.373	0.731	5.1	F_SC_ = 0.25399	<0.0001
Within sampling sites	470	1008.621	2.146	14.97	F_ST_ = 0.85033	<0.0001
Total	489	5107.973	14.33781			

##### Sub‐set GENELAND

**FIGURE 5:**
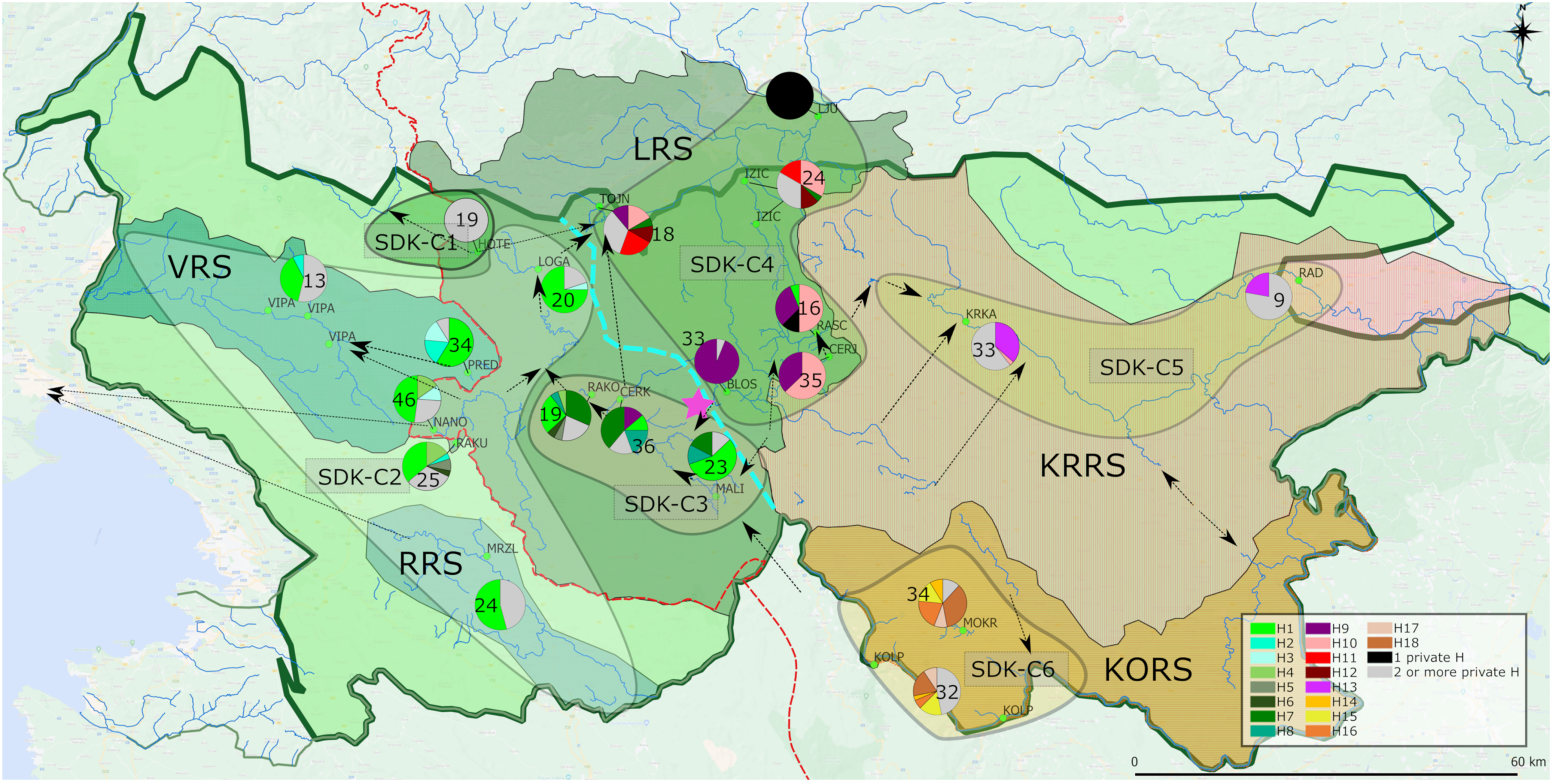
Map showing the sampling sites of *Phoxinus lumaireul* within examined river system in Slovenian Dinaric karst (SDK) and the simplified hydrological underground connections (black arrows) between them. Haplotype frequencies of the *mt* dataset for each sampling site are reported as a pie chart with the number of individuals reported within or next to the chart. One private haplotype is coloured black, while two or more private haplotypes within one sampling site are coloured grey. Population clusters revealed by GENELAND are indicated and named SDK‐C1–SDK‐C8. Dashed red line represents the divide between the Adriatic and Black Sea basins; turquoise dashed line represents the divide within Ljubljanica river system (LRS) between clade 1a and 1c. Pink star shows possible zone of admixture between clades 1a and 1c.

GENELAND analysis of the SDK sampling sites revealed six clusters (SDK‐C1–C6; Figure 5), which were reproduced stably in all 20 runs. Posterior probabilities for each sampling site to belong to a given group are depicted in Figure S3. The clusters comprised SDK‐C1, which includes HOTE (south‐western LRS); SDK‐C2, which contains the VRS and RRS and LOGA, NANO and RAKU from the south‐western LRS. SDK‐C3 included the remaining sites from south‐western LRS RAKO, CERK and MALI. SDK‐C4 contained the north‐eastern LRS BLOS, CERK, RASC, TOJN, IZIC and LJU. SDK‐C5 included the two sampling sites of KRRS KRKA and RAD, while the sixth cluster (SDK‐C6) comprised the sampling sites of KORS KOLP and MOKR.

Results of the AMOVA using the revealed GENELAND clusters in the SDK showed also high genetic differentiation (F_ST_ = 0.85; P ≤ 0.0001). Most (79.94%) of the variation was among clusters, with only 14.97% within sampling sites (P ≤ 0.0001; Table 3).

#### 3.1.6 Divergence timing

A log Bayes factor of >150 was a result that favours our chosen model (log‐normal clock model and birth‐death tree prior) and indicated lack of evidence of the strict clock model and other tree priors (Table S4).

**FIGURE 6:**
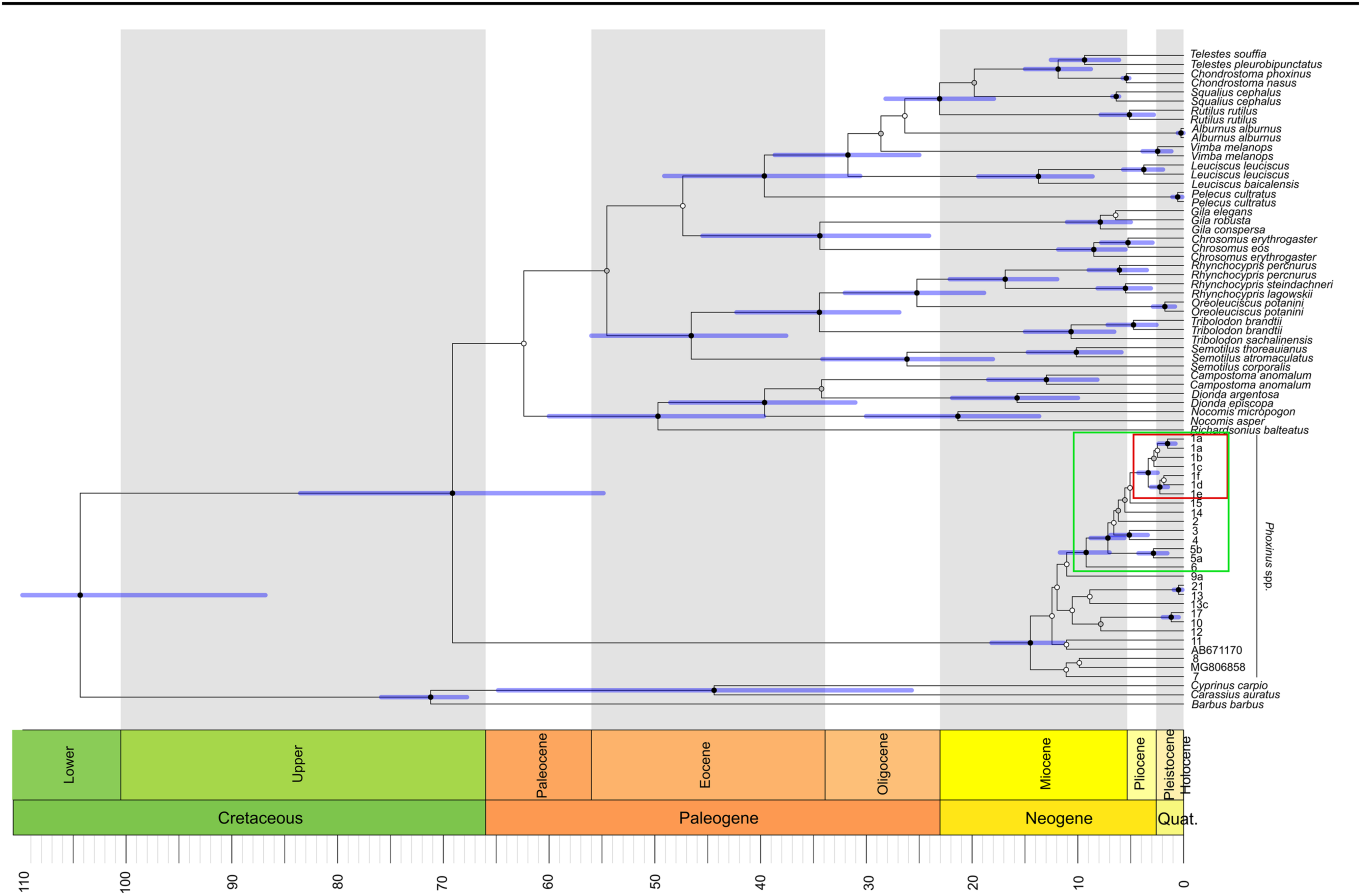
Divergence time estimates within the genus *Phoxinus* and related genera (details are reported in Table S2) based on the mitochondrial marker cytb. *Phoxinus* clades 1–6, 14 and 15 sensu Palandačić et al. (2017) are highlighted in green and designated as Balkan minnows. Clades 1a–c, lineages of *Phoxinus lumaireul* occurring in Dinaric karst of Slovenia, are highlighted in red. Time of splitting is presented by horizontal bars corresponding to a 95% HPD interval. Posterior probabilities (pp) ≥ 0.95 are indicated as black dots, pp ≤ 0.95 and ≥0.75 are grey and pp ≤ 0.75 are white.

According to the divergence timing (measured in millions of years), diversification of the genus *Phoxinus* began in the middle Miocene (95% Highest Posterior Density, HPD: 11.25–18.52; Figure 6). Meanwhile, diversification of the ‘Balkan minnows’ (clades 1–6, 14 and 15 sensu Palandačić et al., 2017) was dated to the middle–late Miocene (95% HPD: 7.05–11.43), when the ancestor of clade 6 (*Phoxinus krkae*, Krka River in Croatia) split from the remaining clades 1–5, 14 and 15. The divergence of clade 5 (*Phoxinus csikii*) from the remaining clades occurred in the late Miocene (95% HPD: 5.51–8.62) and further split into two clades (clades 5a and 5b) in the late Pliocene (95% HPD: 1.61–4.11). The node separating clades 3 and 4 from the remaining clades was not supported, while the split between clades 3 and 4 was dated to the late Miocene–early Pliocene (95% HPD: 3.11–6.82).

Separation of clades mt‐1a‐c and 1d‐f was well supported and placed in the early Pliocene. There was no support for the split within mt‐1a‐c, which supposedly diverged at the end of the Pliocene to Pleistocene. Clade 1a split into an Italian and a Slovenian lineage in the late Pleistocene (node well supported with 95% HPD: 0.78–2.32).

### 3.2 Nuclear DNA

#### 3.2.1 DNA amplification, cloning, sequencing and alignment

The amplification and cloning of 208 individuals resulted in 262 *RPS7* sequences, with a length of 940 bp and where 90 different alleles were detected. The *RPS7* alignment displayed 164 polymorphic sites, indicating the usefulness of the marker for studies of closely related populations and species.

#### 3.2.2 Phylogenetic tree reconstruction

The constructed phylogenetic tree lacked support for the clades (data not shown), and thus, only the allele network (below) is presented, where the structure is denoted with nc‐groups.

#### 3.2.3 Median‐joining network, population diversity indices

**FIGURE 7:**
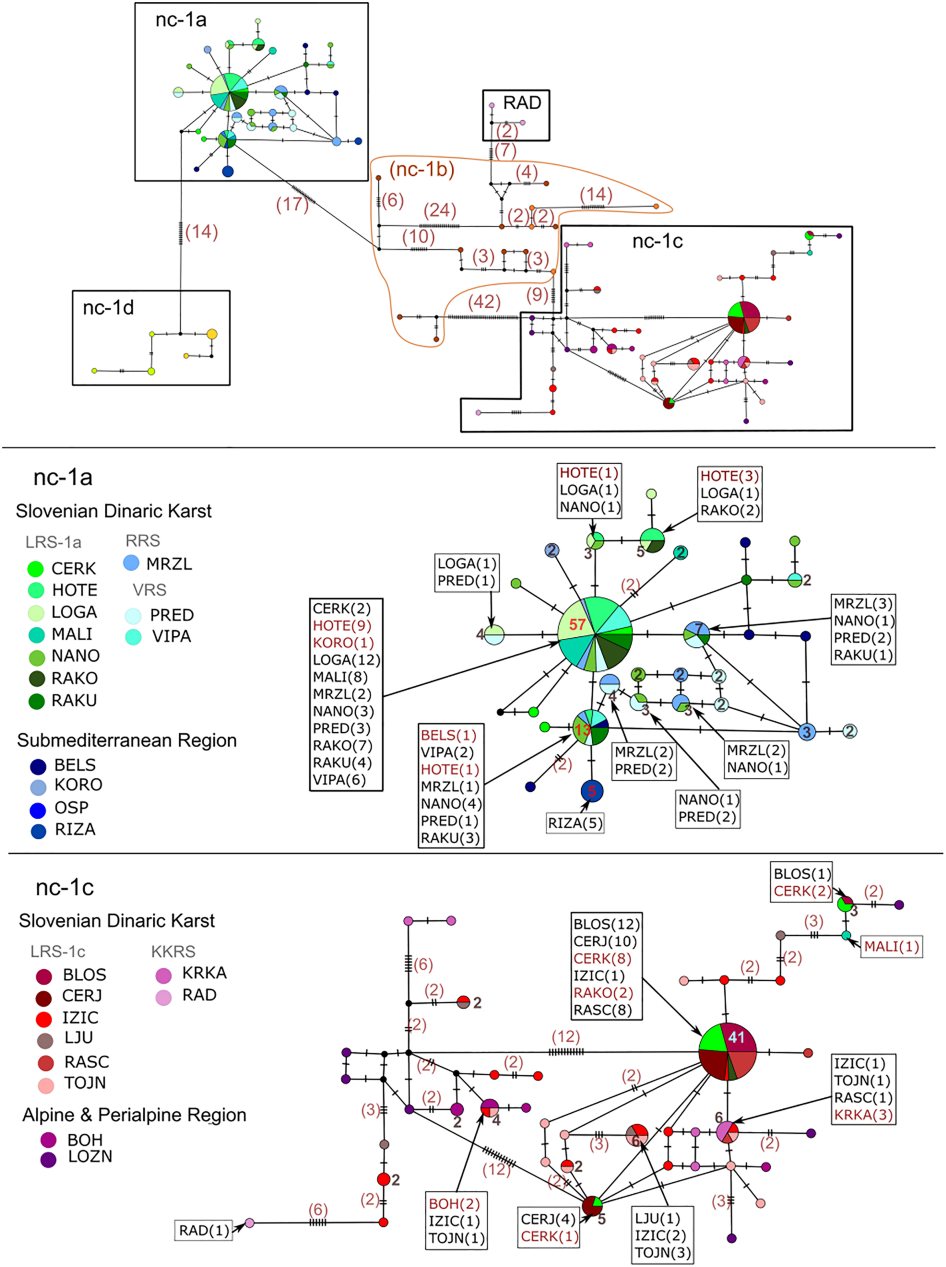
Overview of nuclear (*RPS7*) median‐joining haplotype network of *Phoxinus lumaireul* in Slovenia. The dataset includes sequences generated in this study and sequences obtained from previous studies (Palandačić et al., 2015, 2017). A total of 90 alleles are present. Clades nc‐1a and nc‐1c are presented in a higher magnification. Number of individuals (when N ≥ 1) contributing to each allele are reported next to the haplotype. Mutation steps are indicated with vertical lines. Black dots represent haplotypes missing in the sampling study. Abbreviations of localities are explained in Table 1.

In the calculated allele network, specimens that clustered with clades mt‐1a and mt‐1d were clearly separated, while those that clustered with mt‐1b were ungrouped though scattered around the centre of the network (Figure 7). Individuals from the non‐monophyletic group mt‐1c formed a separate part of the *RPS7* network.

**Group nc‐1a** comprised 29 different alleles (*Hd* = 0.803) and a considerably low level of nucleotide diversity (*π* = 0.0019; Table 4). The dominant allele group included individuals from SDK sampling sites of the south‐western LRS (CERK, MALI, NANO, LOGA, RAKU and HOTE), RRS (MRZL) and VRS (VIPA and PRED), as well as from the SMR sampling site KORO. Another allele group was formed with the SDK sampling sites from the south‐western LRS (RAKU, NANO and HOTE) and VRS (VIPA and PRED) and also included individuals from the SMR sample BELS. There were several additional allele groups sharing individuals from the LRS, RRS and VRS sampling sites (Figure 3). Of the non‐SDK samples, only RIZA formed an exclusive allele group. Notably, HOTE clustered with nc‐1a but did not form its own allele group. Only three CERK individuals (with four different alleles; two homozygotes and one heterozygote individual) were found in nc‐1a, while the others clustered with nc‐1c.

**TABLE 4:** Genetic diversity parameters of the RPS7 dataset

ID	N	*Hd*	*π*	*S*	*k*	No. of sequences	No. of individuals
Alpine & Prealpine Regions							
BOH	4	0.867	0.0067	17	2.88	6	5
LOZN	6	1	0.01305	24	12.06667	6	5
Slovenian Dinaric Karst							
Ljubljanica river system (LRS)							
BLOS	2	0.154	0.00116	7	1.07692	13	12
CERJ	2	0.44	0.0005	1	0.43956	14	10
CERK	6	0.714	0.01977	47	18.26667	15	11
HOTE	4	0.571	0.00102	3	0.94336	14	11
IZIC	14	0.983	0.01297	32	12	16	11
LOGA	5	0.507	0.00094	4	0.86765	17	16
LJU						4	2
MALI	3	0.473	0.00877	43	8.10909	11	9
NANO	10	0.917	0.00246	8	2.41092	16	12
RAKO	3	0.582	0.01451	41	13.41818	12	11
RAKU	4	0.75	0.00102	3	0.94444	9	8
RASC	3	0.378	0.00043	2	0.4	10	10
TOJN	10	0.955	0.00662	24	6.12121	12	11
Reka river system (RRS)							
MRZL	7	0.905	0.0021	4	1.94286	15	7
Vipava river system (VRS)							
PRED	9	0.935	0.00249	6	2.30719	18	12
VIPA	3	0.556	0.0009	3	0.8333	9	7
Kolpa river system (KORS)							
KOLP	9	1	0.03905	86	36.08333	9	7
MOKR	4	1	0.02468	43	22.8333	4	4
Krka river system (KRRS)							
KRKA	5	0.857	0.01196	7	11.04762	7	6
RAD	3	1	0.02013	26	17.333333	3	2
Submediterranean Region							
BELS	5	1	0.00324	6	2.88	5	4
KORO	2	0.667	0.00072	1	0.66667	3	3
RIZA	1	0	0	0	0	5	5
Subpannonian Region							
PTU	2	0.4	0.00086	2	0.8	5	4
SCA	3	0.833	0.0027	5	2.5	4	3
nc‐1a	29	0.803	0.0019	19	1.809	134	
nc‐1b	16	1	0.0364	101	33.58	16	
nc‐1c	40	0.834	0.00816	46	7.5443	103	
nc‐1d	4	0.81	0.0034	7	3.143	7	
Summary	90	0.923	0.02561	164	23.58676	262	208

Abbreviations: π, nucleotide diversity; Hd, haplotype diversity; k, mean number of pairwise differences; N, number of haplotypes; S, number of polymorphic sites.

Allele frequencies in the SDK are shown in Figure S4. The most common allele in nc‐1a (H‐n1) is highlighted in light green and was present in all sampling sites of the SDK.

**Group nc‐1b**. As reported above, individuals that clustered to the clade mt‐1b were not clearly separated in the nuclear network. The group nc‐1b consisted of single alleles that were separated by up to 42 mutational steps (Figure 3; population parameters *Hd* = 1, *π* = 0.0364), while two alleles belonging to the sampling site RAD (based on mt‐DNA belonged to mt‐1c) also clustered to this part of the network (highlighted in Figure 3).

**Group nc‐1c**. In total, 40 different alleles with 46 polymorphic sites were distinguished within the part of the network that represented group nc‐1c (Table 4). This group exhibited large *Hd* (*Hd* = 0.834) and large nucleotide diversity (*π* = 0.00816). The dominant group was formed by alleles from individuals from the north‐eastern LRS (BLOS, CERK, RAKO, IZIC, CERJ and RASC). An allele group was shared between CERJ + CERK, while other mixed allele groups were formed by LJU + IZIC + TOJN, IZIC + KRKA + RASC + TOJN and BOH + IZIC + TOJN. None of the Alpine and Prealpine sampling sites, which clustered to group mt‐1c and formed exclusive haplogroups BOH and LOZN, formed exclusive allele groups.

Three individuals exhibiting admixture between nc‐1a and nc‐1c were detected and where one allele clustered to nc‐1a and the other to nc‐1c. These individuals came from sampling sites in the south‐western LRS: CERK, RAKO and MALI. There was a high frequency of allele H‐n11 (among sampling sites BLOS, CERJ and RASC) in group nc‐1c, and the most common allele among north‐eastern Ljubljanica sampling sites (IZIC, LJU, TOJN) was allele H‐n17 (Figure S4). KRKA and RAD both possessed a large number of private alleles.

**Group nc‐1d** comprised five alleles only (*Hd* = 0.81; *π *= 0.0364) and was separated by 14 mutational steps from nc‐1a. DNA amplification and sequencing failed for sampling site RAT.

#### 3.2.4 Pairwise distance matrix

3.2.4.1

The p‐distances between groups detected by nc‐DNA analysis were considerably larger than by mt‐DNA analysis (Figure 3a,b). Within nc‐1a, the distances were quite small (0–0.05%), with the exceptions of sampling sites MALI, RAKO, CERK, which exhibited greater distances to the remaining sites from nc‐1a (mostly 0.05–1%, though with CERK up to 3–4%). The distances within nc‐1c ranged from 0% to 2% (Figure 3b). KOLP and MOKR differed strongly from the remaining sites, with differences of up to 6% (with mean distances 3–4%). Group nc‐1d showed very small distances (1–3%) from group nc‐1a.

## 4 DISCUSSION

In the present study, three possible scenarios explaining the complex genetic structure of *Phoxinus* minnows in SDK were considered: (1) ongoing geneflow through underground connections, (2) legacies of the past hydrological network and (3) anthropogenic translocations. The results suggest that the first two scenarios have played a major role, while the third has not had a profound effect on the genetic composition of minnows in karst, though this scenario cannot be excluded. Support for the first scenario is found in the mitochondrial genetic structure of the samples from SDK, which is characterized by numerous mutual underground water connections, and that exhibit greater genetic connectivity in comparison to hydrologically isolated reference sampling sites (Alpine and Prealpine, Submediterranean, SPRs) (Figures 2 and 5). Further, within SDK clade mt‐1a sampling sites, among Reka, Vipava and south‐western LRS, extensive mt haplotype sharing was observed (Figure 5, SDK‐C2 and C3, H1 haplotype, denoted in bright green). Finally, the range of Adriatic haplotypes (1a) compared with Black Sea haplotypes (1c) did not correspond to the Adriatic–Black Sea basin divide but was shifted northwards in this area (Figure 5, red dashed line and bright blue dashed line, respectively). Evidence against the first scenario is provided by the genetic barrier found between the south‐western and north‐eastern LRS sampling sites, which share several direct subterranean connections. Regardless, they did not cluster to the same clade (phylogenetic tree, haplotype network) or cluster (full or sub‐set GENELAND) and showed only very limited haplotype sharing (e.g., CERK). The genetic isolation of the remaining two major SDK RS, Kolpa and Krka, support the second scenario. Both RS formed their own genetic clusters (SDK‐C5, SDK‐C6). The third scenario—human introductions—might be reflected in the genetic composition of the north‐eastern LRS, which consists of several haplogroups rather than a monophyletic clade. When considering the nuclear marker (Figure 7), the genetic make‐up was less structured and included allele sharing between SDK and other sampling sites. Together with the conducted timing analysis, this could point to a relatively recent divergence among populations, consistent with the beginning of karstification in this area (Figure 6). While further sampling and more in‐depth analysis of additional genetic markers is required, the present study offers a valuable insight into the factors that have shaped the genetic composition of populations of minnows dwelling in a complex karst aquifer.

### 4.1 Genetic structure as a consequence of ongoing geneflow through underground connections

The SDK and reference sampling sites within the mt‐1a clade were typically well separated genetically, a finding supported by all the mt‐DNA analyses that were conducted (haplotype network, Figure 2; phylogenetic tree, Figure S2; pairwise distance, Figure 3; GENELAND, Figure 4). Moreover, while reference sampling sites each clustered to their own mt‐haplogroups, the SDK samples often shared haplotypes and formed one coherent population, with AMOVA indicating low genetic variation among the sampling sites within the designated clusters (7%). This finding is supported also by the considerably high levels of Hd and rather low levels of nucleotide diversity present in small streams (e.g., PRED, NANO, RAKU, RAKO), pointing to the existence of a single cohesive hydrological network in which the high levels of genetic diversity are maintained through recurring dispersal and population interchange. Thus, the detected genetic structure partially reflects underlying hydrological connections, with the most prominent example being the area within the LRS (sampling sites NANO, RAKU), which is characterized by hydrological bifurcation, where water is flowing to two different water basins (Pivka valley, Habič, 1989). Indeed, here, extensive haplotype sharing and population connectivity were detected between the south‐western Ljubljanica, Vipava and RRS sampling sites, and instead of matching the Adriatic–Black Sea basin divide running through this area, the genetic delimitation between mt‐1a and mt‐1c was shifted to the north‐east (blue dotted line in Figure 5). The discrepancy between the distribution of *Phoxinus* genetic lineages and river–sea drainages has been observed previously (Palandačić et al., 2015; Vučić et al., 2018), most frequently in karst areas, where Palandačić et al. (2020) suggested the difference had a natural origin. However, even though shifted, the genetic divide between south‐western (mt‐1a, full GENELAND C3) and north‐eastern (mt‐1c, full GENELAND C5; Figure 5) LRS sampling sites still exists, despite numerous underground water connections between them (Figures 1b and 6). Further expansion of clade 1a to the north‐east into the Black Sea basin was possibly obstructed by hydrogeological barriers between LOGA, HOTE and the springs of Ljubljanica River (TOJN; Grabovšek & Turk, 2010; Blatnik, 2020), as well as the barrier to flow between the Idrija Fault Zone (sampling sites NANO, RAKU, MRZL, PRED, CERK, RAKO, MALI) and Ljubljana Basin (TOJN; Kaufman et al., 2020). There are few other studies on fishes with a dense sampling set in this area, though in the case of bullheads (Bravničar et al., 2021), the genetic structure follows the Adriatic–Black Sea basin divide, with one species populating the Adriatic and other the Black Sea basin. When comparing the two genera, *Phoxinus* seems to be much more adaptable to different environmental conditions, while *Cottus* is stenotopic and sedentary. Thus, the latter might have reduced dispersal abilities, and the genetic structure reflects hydrological conditions in the area before the beginning of karstification. A similar genetic pattern (albeit with reduced sampling) consistent with the Adriatic–Black Sea basin divide has been observed in chub (*Squalius* sp.; Bogutskaya & Zupančič, 2010) and *Telestes* (Ketmaier et al., 2004). In *Phoxinus*, there is possibly some interchange between the north‐east and south‐west LRS sampling sites, as haplotype sharing has been identified between ‘border’ sampling sites CERK, MALI, RAKO and IZIC + TOJN. However, the discordance between the mt and nc analysis of the MALI and RAKO sampling sites, which points to incomplete lineage sorting, suggests a recent isolation of populations with no subsequent gene flow, with the populations slowly drifting apart and reflecting the past hydrological connections.

### 4.2 Genetic structure as a legacy of a past hydrological network

The two major RS Krka and Kolpa form their own mt‐clusters (C4 and C6, Figure 4; SDK‐C5 and SDK‐C6, Figure 5) and in the case of KORS even its own clade (mt‐1b), suggesting vicariance induced by hydrological fragmentation rather than ongoing migration through underground links. While the difference might relate to different hydrological conditions (e.g., greater flow velocities and water volumes in rivers with fewer underground connections compared with small streams in the LRS with numerous subterranean water connections), the genetic isolation of the Krka and KORS has also been noticed in other fish (e.g., *Cottus* sp. in Kolpa, Bravničar et al., 2021; *Hucho hucho*, Snoj et al., 2022; and *Thymallus thymallus* [unpublished data]) and in invertebrate species (Kolpa: Trontelj et al., 2005; Klobučar et al., 2013; Ivković & Plant, 2015; and Krka: Verovnik et al., 2004). The timing analysis suggests a split between mt‐1a–c and mt‐1d–f within *P. lumaireul* to the middle or late Pliocene, with the start of genetic isolation coinciding with the beginning of the karstification process (Trontelj et al., 2007). While the dating of the further splits (mt‐1a to mt‐1c) is unreliable due to low levels of support at the nodes, it is possible that it took place following the split of mt‐1d at the beginning of, or later during, the Pleistocene. Meanwhile, the polytomous relationship (Figure S2) supports this suggestion, with diversification most probably occurring within a relatively short period (3–5 MYA), consistent also with the genetic structure being influenced by the onset of karstification proposed for stone crayfish in the northern–central Dinarides (Klobučar et al., 2013). Likewise, specific water conditions, where karst streams function as ‘freshwater islands’, are thus considered an isolating factor, proposed also in Previšić et al. (2014) and Bilandžija et al. (2013). In fish, extreme genetic structuring was found in *Salmo marmoratus* in small streams of SDK, explained by impassable barriers preventing immigration into the main RS (Fumagalli et al., 2002). Furthermore, hydrologic isolation was used to explain the genetic structure of karst‐dwelling fishes such as *Telestes croaticus* (Marčić et al., 2011) and *Cobitis* sp. (Buj et al., 2014).

### 4.3 Genetic structure as a consequence of anthropogenic translocations

Despite numerous studies reporting the impact of human introduction on the dispersal patterns of *Phoxinus* species (Corral‐Lou et al., 2019; Garcia‐Raventós et al., 2020; Knebelsberger et al., 2015; Miró & Ventura, 2015; Museth et al., 2007), its influence on the dispersal of *P. lumaireul* in SDK seems to be minimal. Nevertheless, there are some cases that point to anthropogenic translocations. For example, MOKR, a small sinking stream within KORS (mt‐1b), forms a single population with KOLP (GENELAND C6 and SDK‐C6) despite the lack of underground connections. It is possible, therefore, that minnows have been introduced here, even though MOKR appears genetically very heterogeneous (*Hd* = 0.815) and also exhibits three unique haplotypes, findings that are incongruent with a recently introduced population. Similarly, anthropogenic influences might explain the heterogenous genetic composition of *Phoxinus* at the sampling sites IZIC and TOJN (Figure 2, mt‐1c), where there are two main haplogroups more than 25 mutational steps apart, several additional haplogroups shared by both and two specimens (one each) bearing mt‐1a haplotypes. One possible explanation for this pattern is the fire at chemical waste processing plant Kemis in Vrhnika in the year 2017 that killed the fish populations in the spring area of Ljubljanica (TOJN) and that was followed by stocking. Additionally, one mt‐1a specimen bore the most prominent mt‐1a H1 haplotype in RASC, with nc analysis of all three individuals showing no sign of admixture (Figure 3), pointing to a recent introduction of new genetic lineages.

### 4.4 *Phoxinus lumaireul* as a biological tracer

The species *P. lumaireul* is eurytopic and inhabits both karstic and non‐karstic streams in Slovenia and thus seems to be a perfect candidate for assessment of the usefulness of population composition for deciphering a complex underlying aquifer. Certainly, the genetic structure seems to reflect a combination of past and ongoing hydrological connections. Within mt‐1a, haplotype sharing (H1, bright green in Figure 5) between the (i) southwestern LRS (PRED, NANO, RAKU, RAKO, CERK, MALI and LOGA), VRS (VIPA) and RRS (MRZL) sampling sites (GENELAND C3, Figure 4) was observed, as well as between all these listed sampling sites, and (ii) RAKO, CERK and MALI. According to full GENELAND analysis, all sampling sites were also designated to C3, though finer‐scale analysis showed the first group clustered to SDK‐C2 while the second formed SDK‐C3 (Figure 5). Hydrological analysis indicates that the SDK‐C2 Ljubljanica and VRS sampling sites are connected by permanent water connections (Figures 1 and S1, Petrič et al., 2020), while RRS at present seems to be only occasionally or indirectly connected, or both, to LRS (Figure S1), though they were linked several times during the Pleistocene (Habič, 1989). According to Hartl and Clark (2007), occasional gene flow, with a few migrants per generation, is enough to maintain a population in genetic equilibrium, suggested also for karst‐dwelling fish species *Delminichthys adspersus* by Palandačić, Matschiner, et al. (2012). In contrast, SDK‐C3 seems to be permanently connected to farther distant LOGA (SDK‐C2) sampling site through the Planina cave and Unica River but exhibits partial genetic isolation. The reason for this situation might be the temporary flow diversion along the Rak (RAKO) branch detected by Kaufman et al. (2020) that maintains some, though incomplete, population connectivity. Meanwhile, within the northeast LRS (RASC, CERJ, BLOS, IZIC and TOJN, SDK‐C4), the genetic structure is in line with the underground connections between RASC and CERJ. However, there are no known subterranean water connections between the remaining sampling sites. Together with the considerably large genetic distances among these sampling sites (up to 1.5%), the results, which still indicate the sampling sites as supporting a single population, possibly point to the past hydrological situation rather than recent gene flow. Similarly, it seems that the population at the sampling site HOTE, which exhibits incomplete lineage sorting when comparing the results of mt and nc analysis, was previously connected to the LOGA population, while at the present, it is isolated, despite the underground connections between these sites. Possibly, these water connections no longer allow the passage of *Phoxinus*, but the result might also point to a minimal effect of the underground links upon their population structure. Thus, while the genetic composition of *P. lumaireul* has helped to shed some light on past and previous hydrology of the karst area, a more in‐depth analysis with further reference sampling sites and nuclear markers, such as microsatellites, is needed to assess the usefulness of this species as a biological tracer. Furthermore, a detailed comparison with other fishes and other organisms is required to draw conclusions on how the biology of a species influences its dispersal potential in karst aquifers.

## Supporting information




**Figure S1.** Map showing all main, side and unreliable groundwater flow connections in Slovenian Dinaric Karst (SDK), evaluated by tracer tests and digitalized by Petrič et al. (2020). All sampling points are included, red line indicates the border between Adriatic and Black Sea basins. Main river systems in SDK are depicted. Green bold line indicates the border of SDK.

**Figure S2.** Trees based on a collapsed alignment of concatenated COI and cytb sequences of *Phoxinus lumaireul*. A total of 134 unique haplotypes from samples of this study and previous studies (Palandačić et al., 2015; Palandačić et al., 2017; Table S1 for details) are distinguishable in three clades (mt‐1a, −1b, −1d) and one group (mt‐1c). Sample localities are given with abbreviations (explained in Table 1) **A.** Bayesian Inference (BI) tree. Branches with a Bayesian probability ≤0.95 are dashed. **B.** Maximum likelihood tree. Branches with a bootstrap support ≤0.9 are dashed.

**Figure S3.** Population Bayesian cluster analysis (GENELAND). Only sampling sites of Slovenian Dinaric Karst (SDK) were analysed (sub‐set GENELAND). Maps show the geographic distribution of samples (black points) **A.** Map of cluster membership for each sampling site (K = 6). **B.** Relative posterior probability of belonging to each of the six inferred clusters. Darker colour reflects higher posterior probability.

**Figure S4.** Haplotype frequencies of the nc dataset for each sampling site. Haplotype frequencies are given as a pie chart and the number of alleles and number of individuals in brackets is reported within or next to the pie chart. One private haplotype is coloured black, while two or more private haplotypes within one sampling site are coloured grey. Dashed red line represents the divide between the Adriatic and Black Sea basins; turquoise dashed line represents the divide within the Ljubljanica river system (LRS) between clades 1a and 1c. Pink star shows possible zone of admixture between clades 1a and 1c.

**Table S1.** All examined specimens with GenBank accession numbers from this study and previous studies (Palandačić et al. 2015, 2017). Sampling sites with coordinates, drainage basins, clades, and sampling dates are given.

**Table S2.** All samples with GenBank accession numbers and references included in the divergence timing analysis.
**Table S3.** Marginal likelihoods with standard deviation of four models tested with nested sampling for divergence time analysis (further information in Material & methods section). M1 is the model used for further analysis. Abbreviations: uclc, uncorrelated relaxed log‐normal clock; strict, strict clock; BD, Birth‐death model; Yule, Yule model; BS, Coalescent Bayesian skyline.

## Data Availability

The data that support the findings of this study are available in the supplementary material of this article. Sequence data (sequences generated in the course of the present study as well as previously published once) are available online in the NCBI Database.
