# Supplementary figures and images for: The minnow Phoxinus lumaireul (Leuciscidae) shifts the Adriatic–Black Sea basin divide in the north‐western Dinaric Karst region
Source: Ecohydrology. 2022 Jul 13;15(6):e2449. doi: 10.1002/eco.2449 (PMC9539529; doi:10.1002/eco.2449)

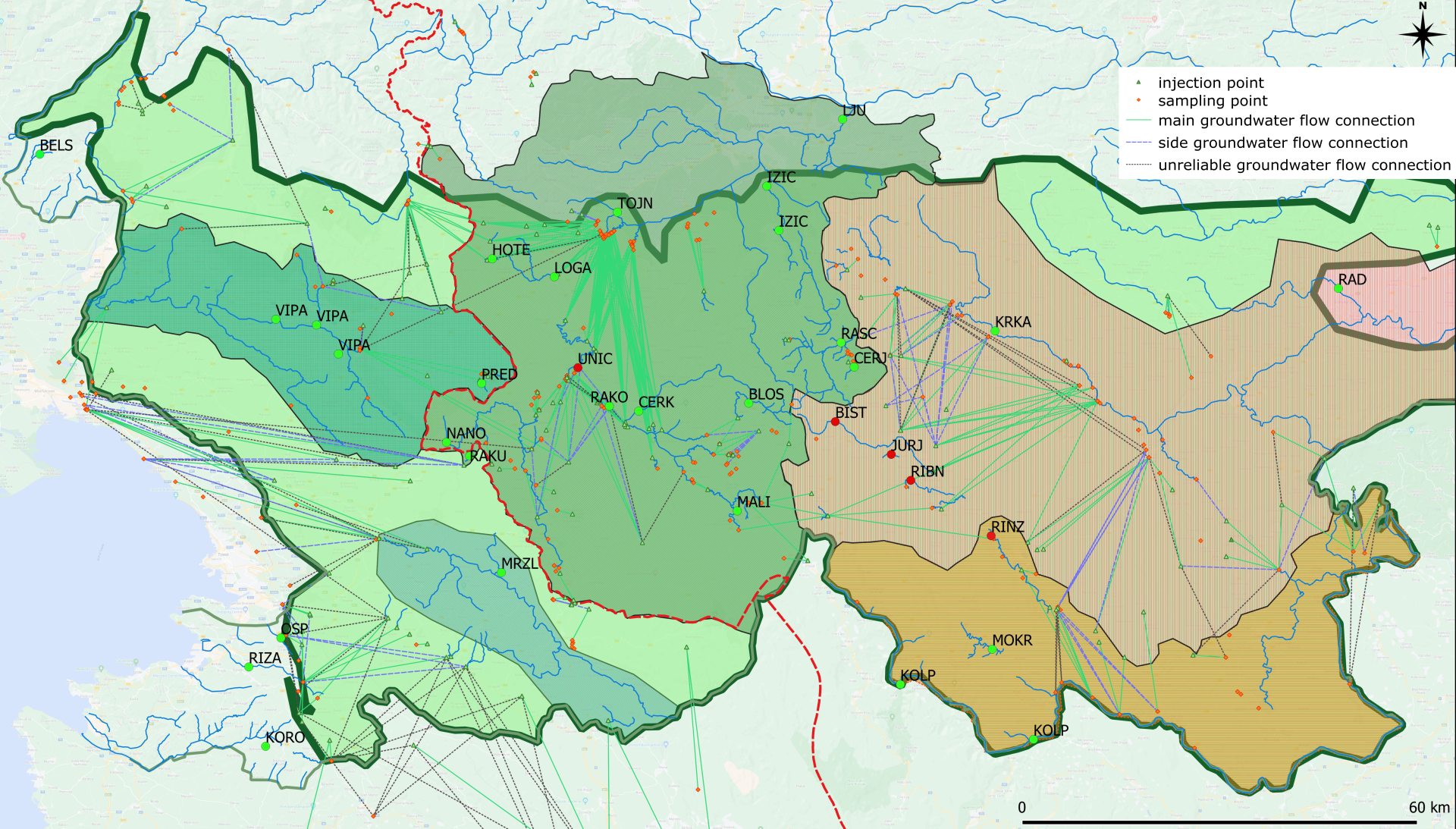

Supplement: Supplementary file 1 — Figure S1. Map showing all main, side and unreliable groundwater flow connections in Slovenian Dinaric Karst (SDK), evaluated by tracer tests and digitalized by Petrič et al. (2020). All sampling points are included, red line indicates the border between Adriatic and Black Sea basins. Main river systems in SDK are depicted. Green bold line indicates the border of SDK. [file ECO-15-e2449-s006.tif]

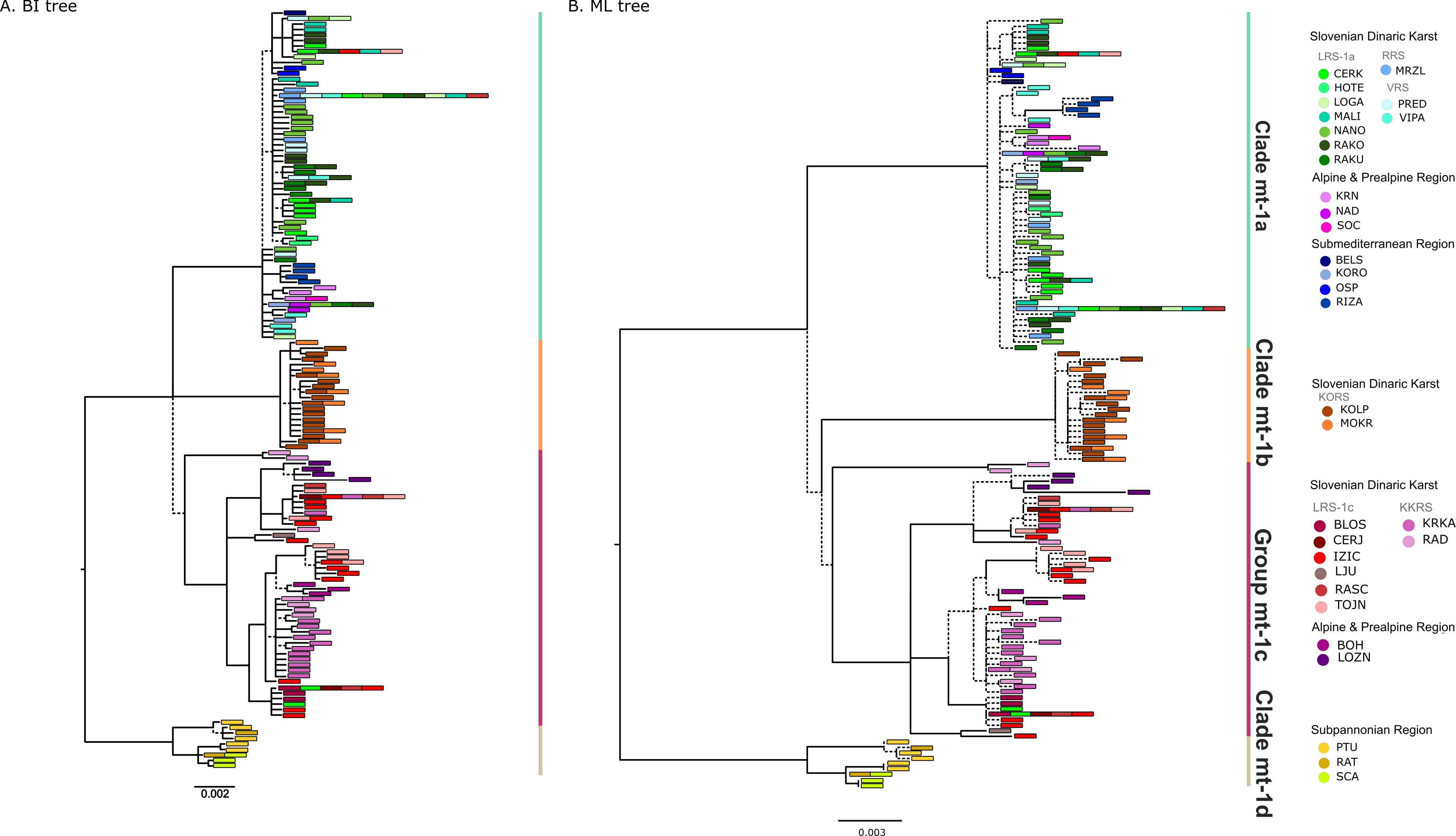

Supplement: Supplementary file 2 — Figure S2. Trees based on a collapsed alignment of concatenated COI and cytb sequences of Phoxinus lumaireul. A total of 134 unique haplotypes from samples of this study and previous studies (Palandačić et al., 2015; Palandačić et al., 2017; Table S1 for details) are distinguishable in three clades (mt‐1a, −1b, −1d) and one group (mt‐1c). Sample localities are given with abbreviations (explained in Table 1) A. Bayesian Inference (BI) tree. Branches with a Bayesian probability ≤0.95 are dashed. B. Maximum likelihood tree. Branches with a bootstrap support ≤0.9 are dashed. [file ECO-15-e2449-s005.tif]

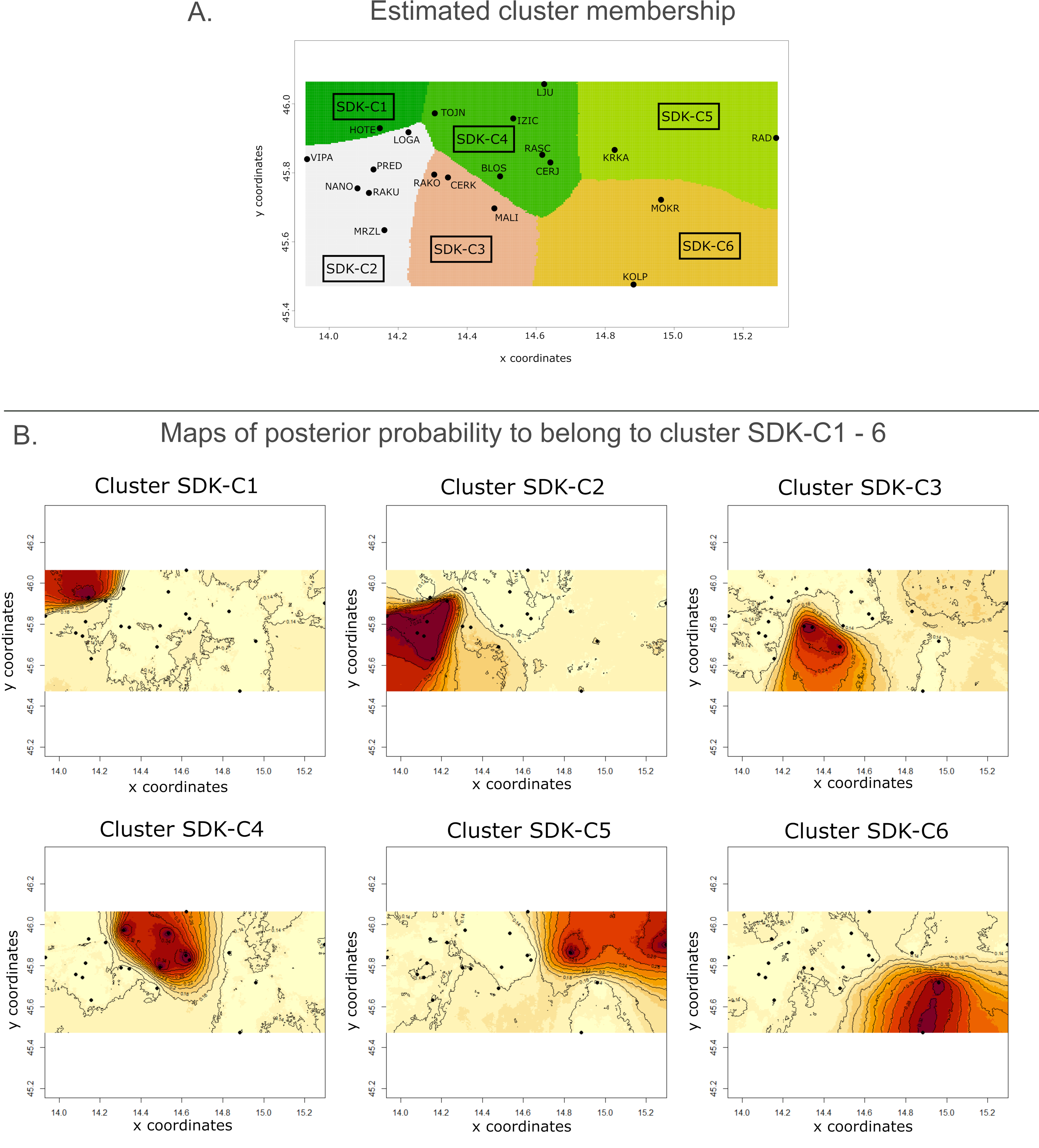

Supplement: Supplementary file 3 — Figure S3. Population Bayesian cluster analysis (GENELAND). Only sampling sites of Slovenian Dinaric Karst (SDK) were analysed (sub‐set GENELAND). Maps show the geographic distribution of samples (black points) A. Map of cluster membership for each sampling site (K = 6). B. Relative posterior probability of belonging to each of the six inferred clusters. Darker colour reflects higher posterior probability. [file ECO-15-e2449-s001.tif]

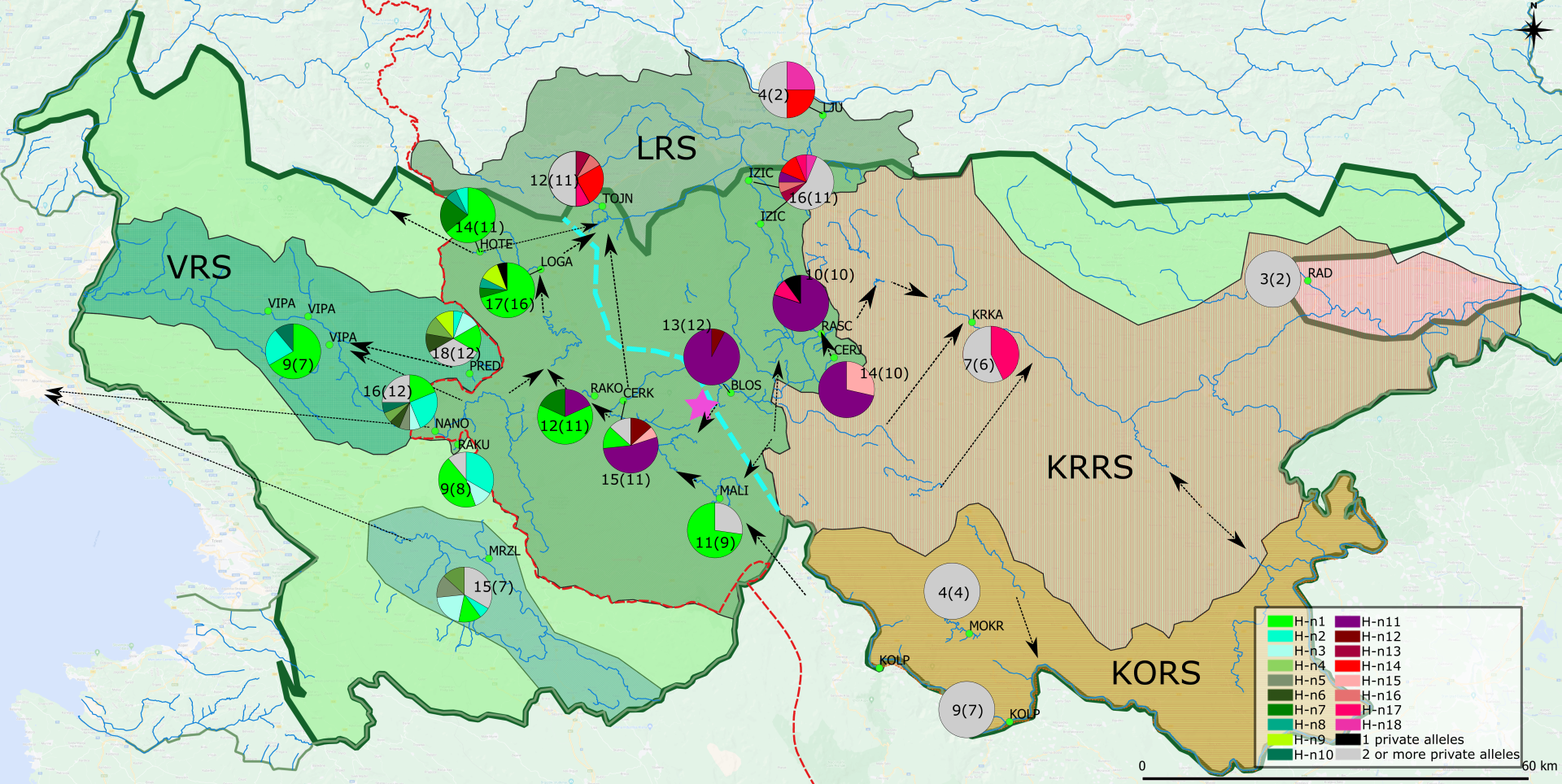

Supplement: Supplementary file 4 — Figure S4. Haplotype frequencies of the nc dataset for each sampling site. Haplotype frequencies are given as a pie chart and the number of alleles and number of individuals in brackets is reported within or next to the pie chart. One private haplotype is coloured black, while two or more private haplotypes within one sampling site are coloured grey. Dashed red line represents the divide between the Adriatic and Black Sea basins; turquoise dashed line represents the divide within the Ljubljanica river system (LRS) between clades 1a and 1c. Pink star shows possible zone of admixture between clades 1a and 1c. [file ECO-15-e2449-s003.tif]
