# Supplementary material for: The minnow Phoxinus lumaireul (Leuciscidae) shifts the Adriatic–Black Sea basin divide in the north‐western Dinaric Karst region
Source: Ecohydrology. 2022 Jul 13;15(6):e2449. doi: 10.1002/eco.2449 (PMC9539529; doi:10.1002/eco.2449)
Supplement: Supplementary file 6 — Table S2. All samples with GenBank accession numbers and references included in the divergence timing analysis. Table S3. Marginal likelihoods with standard deviation of four models tested with nested sampling for divergence time analysis (further information in Material & methods section). M1 is the model used for further analysis. Abbreviations: uclc, uncorrelated relaxed log‐normal clock; strict, strict clock; BD, Birth‐death model; Yule, Yule model; BS, Coalescent Bayesian skyline. [file ECO-15-e2449-s004.docx]

**The minnow *Phoxinus lumaireul* (Leuciscidae) shifts the Adriatic–Black Sea basin divide in the northwestern Dinaric Karst region**

Susanne Reier, Luise Kruckenhauser, Ales Snoj, Peter Trontelj, Anja Palandačić

**Supplementary material**

1. **Detailed description of the study area**
   1. **River systems in Slovenian Dinaric Karst**
      1. *Ljubljanica river system*

Ljubljanica RS includes several karst poljes developed along the Idrija Fault connected by eight sinking streams under different names.

1. Trbuhovica first appears in the field of Prezidsko polje in Croatia and disappears in Babno polje. No *Phoxinus* are recorded according to BIOSWEB (Biological database of the Fisheries Research Institute of Slovenia, <http://www.biosweb.org/index.php?task=map&tid=4026>, accessed 30.03.2021)
2. Obrh flows on Loško polje, disappears and reoccurs on the Cerknica polje. *Phoxinus* were sampled in Mali Obrh (MALI).
3. Stržen is located in Cerknica polje, where the Cerkniščica (*Phoxinus* sampled there at CERK) runs from the north (Blatnik 2020). It is also connected underground to the Bloke Plateau (Kogovšek, Prelovšek, and Petrič 2008), where river Bloščica (BLOS) was sampled.
4. Rak drains through Rakov Škocjan polje (*Phoxinus* sampled at RAKO) and disappears in Planinska Jama Cave, where it meets River Pivka (Perko et al. 2020).
5. Pivka flows through Pivka basin and Postojna Cave. In Pivka no *Phoxinus* were recorded; thus fish were collected in its tributary Nanoščica (NANO) and a sinking stream flowing in the vicinity: Rakulik (RAKU).
6. Unica is formed where Pivka and Rak join in Planinska Jama Cave; it flows along Planinsko polje (Blatnik 2020). According to BIOSWEB, *Phoxinus* were recorded in Unica only once, in the year 1984, while during the collecting for this study, regardless of extensive efforts, none were observed.
7. Logaščica partly involves the sinking system of Rovte, Hotenjsko podolje (Hotenka (HOTE) was sampled) and Logaško polje (Blatnik 2020). Phoxinus were sampled in Logaščica stream (LOGA).
8. Ljubljanica springs near Vrhnika (confluence Tojnica (TOJN) was sampled), runs through the Ljubljana marshes (confluence Ižica (IZIC) was sampled), and flows into the Sava at Podgrad (sampling site LJU, located outside of the SDK territory).

On the edge of the Ljubljanica RS, near the Krka river system, rivers Rašića (RASC) and Cereja (CERJ) were sampled. While they are connected underground, tracing tests show that Rašića is also indirectly linked to Mali Obrh (MALI) through its tributary Kozmanjka (Figure 1B & Figure S1 for more details).

Although the geographic position of the Ljubljanica RS is in the Black Sea basin, it represents a bifurcation of the Adriatic–Black Sea basins: some streams of Pivka basin (NANO) flow underground to the rivers Timavo and Vipava, while Hotenka (HOTE) is connected underground with Idrijca (Blatnik 2020); (Perko et al. 2020).

- - 1. *Vipava river system*

The main water body of the Vipava RS mostly flows on flysch. *Phoxinus* were sampled in Vipava and some of its tributaries (all pooled: VIPA). Vipava springs in the karst and from there, 14 km to the northwest, it is connected underground to River Lovka, sinking into Predjama Cave (Šebela and Turk 2014). *Phoxinus* were sampled there (PRED). The Vipava RS is located in the Adriatic basin.

- - 1. *Reka river system*

Reka is the longest sinking stream in Slovenia, which flows about 50 km on the surface on impermeable flysch rocks and another 7 km on limestone. At the end of its surface flow it reaches Dinaric Karst and sinks underground in the Škocjanske jame (Škocjan caves) (Gabrovšek and Peric 2006). Its underground course to Timavo (Italy) is around 33 km (Timavo is not inhabited by *Phoxinus*). Reka is represented by only one locality, by its tributary Mrzlek (MRZL). The Reka RS is located on the Adriatic basin.

- - 1. *Krka river system*

The sampling included rivers Krka (KRKA) and Radulja (RAD). In this area, additional sinking streams exist (Bistrica (BIST), Ribnica (RIBN), small stream near Jurjevica (JURJ)), which are connected underground to the collected sampling sites. However, according to BIOSWEB and confirmed at the site, they are not populated with *Phoxinus* (red dots in Figure 1B). RAD is a tributary of Krka and belongs to the non-karstic area of the Krka RS (and zoogeographically to the Subpannonian Region). The Krka RS is located in the Black Sea basin.

- - 1. *Kolpa river system*

Two sampling points (Kolpa, KOLP; Mokri Potok, MOKR) belong to the Kolpa RS, which extends from Slovenia to Croatia, and also includes a small part of Bosnia and Herzegovina. MOKR is a small sinking stream and no subterranean links to surrounding water are known. The sinking stream Rinža (RINZ in Figure 1B), which is connected underground to Kolpa is not inhabited by *Phoxinus* according to BIOSWEB and confirmed at the site (Figure 1B). The Kolpa RS is located in the Black Sea basin.

- 1. **Reference sampling sites**
     1. *Alpine & Prealpine Regions*

There are five sampling sites in Alpine & Prealpine Regions: Krn Lake (KRN, introduced), Nadiža (NAD), and Soča (SOC) in the Adriatic basin and Bohinj Lake (BOH) and Ložnica (LOZN) in the Black Sea basin.

- - 1. *Submediterranean Region*

The Submediterranean Region includes three coastal rivers (Malinska (KORO), Osapska (OSP), Rižana rivers (RIZA)), and the stream Belski Potok (BELS) in the western part of Slovenia near the Italian border.

- - 1. *Subpannonian Region*

Subpannonian Region sampling sites include rivers located in the Drava river system (Drava (PTU)) and the Mura river system (Ratkovski (RAT), Ščavnica stream (SCA)).

1. **Tables**

Table S1. All examined specimens with GenBank accession numbers from this study and previous studies (Palandačić et al. 2015; Palandačić et al. 2017). Sampling sites with coordinates, drainage basins, clades, and sampling dates are given.

##Table S1 separate in Excel sheet

Table S2. All samples with GenBank accession numbers and references included in the divergence timing analysis.

| **Species** | **Clade** | **GenBank** | **Reference** |
| --- | --- | --- | --- |
| *Phoxinus lumaireul* | 1a | KT166578 | Palandačić et al. (2015) |
| *Phoxinus lumaireul* | 1a | # | this study |
| *Phoxinus lumaireul* | 1c | # | this study |
| *Phoxinus lumaireul* | 1b | KT166653 | Palandačić et al. (2015) |
| *Phoxinus lumaireul* | 1d | MF408226 | Palandačić et al. (2017) |
| *Phoxinus lumaireul* | 1e | KT166790 | Palandačić et al. (2015) |
| *Phoxinus lumaireul* | 1f | KT166762 | Palandačić et al. (2015) |
| *Phoxinus sp.* | 2 | KT166621 | Palandačić et al. (2015) |
| *Phoxinus sp.* | 3 | KT166691 | Palandačić et al. (2015) |
| *Phoxinus sp.* | 4 | KT166574 | Palandačić et al. (2015) |
| *Phoxinus sp.* | 5a | KT166612 | Palandačić et al. (2015) |
| *Phoxinus sp.* | 5b | KX673469 | Ramler et al. (2017) |
| *Phoxinus sp.* | 6 | KT166548 | Palandačić et al. (2015) |
| *Phoxinus sp.* | 7 | MN820807 | Palandačić et al. (2017) |
| *Phoxinus sp.* | 8 | KT166678 | Palandačić et al. (2015) |
| *Phoxinus sp.* | 9a | MF408194 | Palandačić et al. (2017) |
| *Phoxinus phoxinus* | 10 | MF408222 | Palandačić et al. (2017) |
| *Phoxinus sp.* | 11 | MG806683 | Schönhuth et al. (2018) |
| *Phoxinus sp.* | 12 | MK787687 | Corral‐Lou et al. (2019) |
| *Phoxinus bigerri* | 13 | HM560122 | Perea et al. (2010) |
| *Phoxinus sp.* | 13c | MG681510 | Vučić et al. (2018) |
| *Phoxinus sp.* | 14 | MG806685 | Schönhuth et al. (2018) |
| *Phoxinus sp.* | 15 | MG806686 | Schönhuth et al. (2018) |
| *Phoxinus sp.* | 17 | EU352213 | Strange and Mayden (2009) |
| *Phoxinus sp.* | 21 | MK787690 | Corral‐Lou et al. (2019) |
| *Phoxinus sp.* | Mongolia | AB671170 | Imoto et al. (2013) |
| *Phoxinus sp.* | Bolshoy | MG806682 | Schönhuth et al. (2018) |
| *Oreoleuciscus potanini* |  | KJ754935 | Chen et al. (2016) |
| *Oreoleuciscus potanini* |  | AB626851 | Imoto et al. (2013) |
| *Tribolodon sachalinensis* |  | AB626856 | Imoto et al. (2013) |
| *Tribolodon brandtii* |  | AB626853 | Imoto et al. (2013) |
| *Tribolodon brandtii* |  | AB626854 | Imoto et al. (2013) |
| *Rhynchocypris percnurus* |  | AP009149 | Imoto et al. (2013) |
| *Rhynchocypris percnurus* |  | NC_008684 | Saitoh et al. (2006) |
| *Rhynchocypris lagowskii* |  | AP009148 | Imoto et al. (2013) |
| *Rhynchocypris lagowskii* |  | AP009147 | Imoto et al. (2013) |
| *Pelecus cultratus* |  | AB239597 | Saitoh et al. (2006) |
| *Pelecus cultratus* |  | AY838938 | Freyhof et al. (2006) |
| *Richardsonius balteatus* |  | AP012106 | Iwasaki et al. (2013) |
| *Campostoma anomalum* |  | DQ536421 | Broughton and Reneau (2006) |
| *Campostoma anomalum* |  | KP013113 | Renshaw et al. (unpublished) |
| *Nocomis micropogon* |  | MH324421 | Schroeter et al. (2020) |
| *Nocomis asper* |  | AP012096 | Iwasaki et al. (2013) |
| *Dionda episcopa* |  | AP012077 | Iwasaki et al. (2013) |
| *Dionda argentosa* |  | JN812352 | Schönhuth et al. (2012) |
| *Chondrostoma nasus* |  | MG806657 | Schönhuth et al. (2018) |
| *Chondrostoma phoxinus* |  | MG806658 | Schönhuth et al. (2018) |
| *Telestes pleurobipunctatus* |  | MG806720 | Schönhuth et al. (2018) |
| *Telestes souffia* |  | MG806721 | Schönhuth et al. (2018) |
| *Rutilus rutilus* |  | AP010775 | Saitoh et al. (2011) |
| *Rutilus rutilus* |  | MG806695 | Schönhuth et al. (2018) |
| *Squalius cephalus* |  | NC031540 | Iwasaki et al. (2013) |
| *Squalius cephalus* |  | MG806701 | Schönhuth et al. (2018) |
| *Alburnus alburnus* |  | AB239593 | Saitoh et al. (2006) |
| *Alburnus alburnus* |  | MG806649 | Schönhuth et al. (2018) |
| *Vimba melanops* |  | MG806725 | Schönhuth et al. (2018) |
| *Vimba melanops* |  | AP011212 | Iwasaki et al. (2013) |
| *Leuciscus leuciscus* |  | MG806667 | Schönhuth et al. (2018) |
| *Leuciscus leuciscus* |  | DQ664302 | Costedoat et al. (2006) |
| *Chrosomus erythrogaster* |  | AP011276 | Imoto et al. (2013) |
| *Chrosomus erythrogaster* |  | AY281055 | Mayden (2002) |
| *Chrosomus eos* |  | AP009151 | Imoto et al. (2013) |
| *Gila elegans* |  | MT364326 | Osborne et al. (2020) |
| *Gila robusta* |  | DQ536424 | Broughton and Reneau (2006) |
| *Gila conspersa* |  | AP009315 | Saitoh et al. (2006) |
| *Semotilus atromaculatus* |  | MG570419 | Schroeter et al. (2019) |
| *Semotilus corporalis* |  | MG806644 | Schönhuth et al. (2018) |
| *Semotilus thoreauianus* |  | MG806645 | Schönhuth et al. (2018) |
| *Cyprinus carpio* |  | NC_001606 | Chang, Huang, and Lo (1994) |
| *Barbus barbus* |  | NC_008654 | Saitoh et al. (2006) |
| *Carassius auratus* |  | KJ874430 | Cheng, Liang, and Sun (2012) |
| *Leuciscus baicalensis* |  | KF673863 | Hu et al. (2015) |

Table S3. Marginal likelihoods with standard deviation of four models tested with nested sampling for divergence time analysis (further information in Material & methods section). M1 is the model used for further analysis. Abbreviations: uclc, uncorrelated relaxed log-normal clock; strict, strict clock; BD, Birth-death model; Yule, Yule model; BS, Coalescent Bayesian skyline.

|  | **M1** | **M2** | **M3** | **M4** |
| --- | --- | --- | --- | --- |
| molecular clock | uclc | strict | uclc | uclc |
| tree prior | BD | BD | Yule | BS |
| marginal likelihood | -16062.639 | -47816.488 | -18347.818 | -18344.599 |
| standard deviation | 3.582 | 1.116 | 4.546 | 4.299 |

1. **References**

Broughton, R. E., & Reneau, P. C. (2006). Spatial Covariation of Mutation and Nonsynonymous Substitution Rates in Vertebrate Mitochondrial Genomes. Molecular Biology and Evolution, 23(8), 1516–1524. https://doi.org/10.1093/molbev/msl013

Chang, Y., Huang, F., & Lo, T. (1994). The complete nucleotide sequence and gene organization of carp *(Cyprinus carpio)* mitochondrial genome. Journal of Molecular Evolution, 38(2), 138–155. https://doi.org/10.1007/BF00166161

Chen, I.-S., Liu, G.-D., Shen, C.-N., & Prokofiev, A. M. (2016). The complete mitochondrial genome of altai osman *Oreoleuciscus humilis* Warpachowski (Cypriniformes, Cyprinidae). Mitochondrial DNA, 27(2), 953–955. https://doi.org/10.3109/19401736.2014.926502

Cheng, L., Liang, L.-Q., & Sun, X.-W. (2012). The complete mitochondrial genome of the crucian carp, *Carassius carassius* (Cypriniformes, Cyprinidae). Mitochondrial DNA, 23(5), 350–351. https://doi.org/10.3109/19401736.2012.690753

Corral‐Lou, A., Perea, S., Aparicio, E., & Doadrio, I. (2019). Phylogeography and species delineation of the genus *Phoxinus* Rafinesque, 1820 (Actinopterygii: Leuciscidae) in the Iberian Peninsula. Journal of Zoological Systematics and Evolutionary Research, 57(4), 926–941. https://doi.org/10.1111/jzs.12320

Costedoat, C., Chappaz, R., Barascud, B., Guillard, O., & Gilles, A. (2006). Heterogeneous colonization pattern of European Cyprinids, as highlighted by the dace complex (Teleostei: Cyprinidae). Molecular Phylogenetics and Evolution, 41(1), 127–148. https://doi.org/10.1016/j.ympev.2006.04.022

Freyhof, J., Lieckfeldt, D., Bogutskaya, N. G., Pitra, C., & Ludwig, A. (2006). Phylogenetic position of the Dalmatian genus *Phoxinellus* and description of the newly proposed genus *Delminichthys* (Teleostei: Cyprinidae). Molecular Phylogenetics and Evolution, 38(2), 416–425. https://doi.org/10.1016/j.ympev.2005.07.024

Hu, S., Niu, J., Xie, P., Liu, C., Karjan, A., Wang, F., & Ma, X. (2015). The complete mitochondrial genome of *Leuciscus leuciscus baicalensis* (Cypriniformes: Cyprinidae). Mitochondrial DNA, 26(5), 751–752. https://doi.org/10.3109/19401736.2013.848353

Imoto, J. M., Saitoh, K., Sasaki, T., Yonezawa, T., Adachi, J., Kartavtsev, Y. P., … Hanzawa, N. (2013). Phylogeny and biogeography of highly diverged freshwater fish species (Leuciscinae, Cyprinidae, Teleostei) inferred from mitochondrial genome analysis. Gene, 514(2), 112–124. https://doi.org/10.1016/j.gene.2012.10.019

Iwasaki, W., Fukunaga, T., Isagozawa, R., Yamada, K., Maeda, Y., Satoh, T. P., … Nishida, M. (2013). MitoFish and MitoAnnotator: A Mitochondrial Genome Database of Fish with an Accurate and Automatic Annotation Pipeline. Molecular Biology and Evolution, 30(11), 2531–2540. https://doi.org/10.1093/molbev/mst141

Mayden, R. L. (2002). Phylogenetic relationships of the enigmatic ornate shiner, *Cyprinella ornata*, a species endemic to Mexico (Teleostei: Cyprinidae). Reviews in Fish Biology and Fisheries, 12(2), 339–347. https://doi.org/10.1023/A:1025006625743

Osborne, M. J., Cameron, A. C., Fitzgerald, B. P., McKitrick, S. A., Paulk, M. R., & Turner, T. F. (2020). The complete mitochondrial genomes of three imperiled cyprinid fishes Bonytail (*Gila elegans*), Rio Grande Silvery Minnow (*Hybognathus amarus*) and Loach Minnow (*Tiaroga cobitis*). Mitochondrial DNA Part B, 5(3), 2368–2370. https://doi.org/10.1080/23802359.2020.1774435

Palandačić, A., Bravničar, J., Zupančič, P., Šanda, R., & Snoj, A. (2015). Molecular data suggest a multispecies complex of *Phoxinus* (Cyprinidae) in the Western Balkan Peninsula. Molecular Phylogenetics and Evolution, 92, 118–123. https://doi.org/10.1016/j.ympev.2015.05.024

Palandačić, A., Naseka, A., Ramler, D., & Ahnelt, H. (2017). Contrasting morphology with molecular data: An approach to revision of species complexes based on the example of European *Phoxinus* (Cyprinidae). BMC Evolutionary Biology, 17(1). https://doi.org/10.1186/s12862-017-1032-x

Perea, S., Böhme, M., Zupančič, P., Freyhof, J., Šanda, R., Özuluğ, M., … Doadrio, I. (2010). Phylogenetic relationships and biogeographical patterns in Circum-Mediterranean subfamily Leuciscinae (Teleostei, Cyprinidae) inferred from both mitochondrial and nuclear data. BMC Evolutionary Biology, 10(1), 265. https://doi.org/10.1186/1471-2148-10-265

Ramler, D., Palandačić, A., Delmastro, G. B., Wanzenböck, J., & Ahnelt, H. (2017). Morphological divergence of lake and stream *Phoxinus* of Northern Italy and the Danube basin based on geometric morphometric analysis. Ecology and Evolution, 7(2), 572–584. https://doi.org/10.1002/ece3.2648

Saitoh, K., Sado, T., Mayden, R. L., Hanzawa, N., Nakamura, K., Nishida, M., & Miya, M. (2006). Mitogenomic Evolution and Interrelationships of the Cypriniformes (Actinopterygii: Ostariophysi): The First Evidence Toward Resolution of Higher-Level Relationships of the World’s Largest Freshwater Fish Clade Based on 59 Whole Mitogenome Sequences. Journal of Molecular Evolution, 63(6), 826–841. https://doi.org/10.1007/s00239-005-0293-y

Schönhuth, S., Hillis, D. M., Neely, D. A., Lozano-Vilano, L., Perdices, A., & Mayden, R. L. (2012). Phylogeny, diversity, and species delimitation of the North American Round-Nosed Minnows (Teleostei: Dionda), as inferred from mitochondrial and nuclear DNA sequences. Molecular Phylogenetics and Evolution, 62(1), 427–446. https://doi.org/10.1016/j.ympev.2011.10.011

Schönhuth, S., Vukić, J., Šanda, R., Yang, L., & Mayden, R. L. (2018). Phylogenetic relationships and classification of the Holarctic family Leuciscidae (Cypriniformes: Cyprinoidei). Molecular Phylogenetics and Evolution, 127, 781–799. https://doi.org/10.1016/j.ympev.2018.06.026

Schroeter, J. C., Maloy, A. P., Rees, C. B., & Bartron, M. L. (2020). Fish mitochondrial genome sequencing: Expanding genetic resources to support species detection and biodiversity monitoring using environmental DNA. Conservation Genetics Resources, 12(3), 433–446. https://doi.org/10.1007/s12686-019-01111-0

Strange, R. M., & Mayden, R. L. (2009). Phylogenetic Relationships and a Revised Taxonomy for North American Cyprinids Currently Assigned to *Phoxinus* (Actinopterygii: Cyprinidae). Copeia, 2009(3), 494–501. https://doi.org/10.1643/CI-07-070

Vučić, M., Jelić, D., Žutinić, P., Grandjean, F., & Jelić, M. (2018). Distribution of Eurasian minnows (*Phoxinus*: Cypriniformes) in the Western Balkans. Knowledge & Management of Aquatic Ecosystems, (419), 11. https://doi.org/10.1051/kmae/2017051
